# Supplementary figures and images for: Understanding the uptake and determinants of prevention of mother-to-child transmission of HIV services in East Africa: Mixed methods systematic review and meta-analysis
Source: PLoS One. 2024 Apr 18;19(4):e0300606. doi: 10.1371/journal.pone.0300606 (PMC11025786; doi:10.1371/journal.pone.0300606)

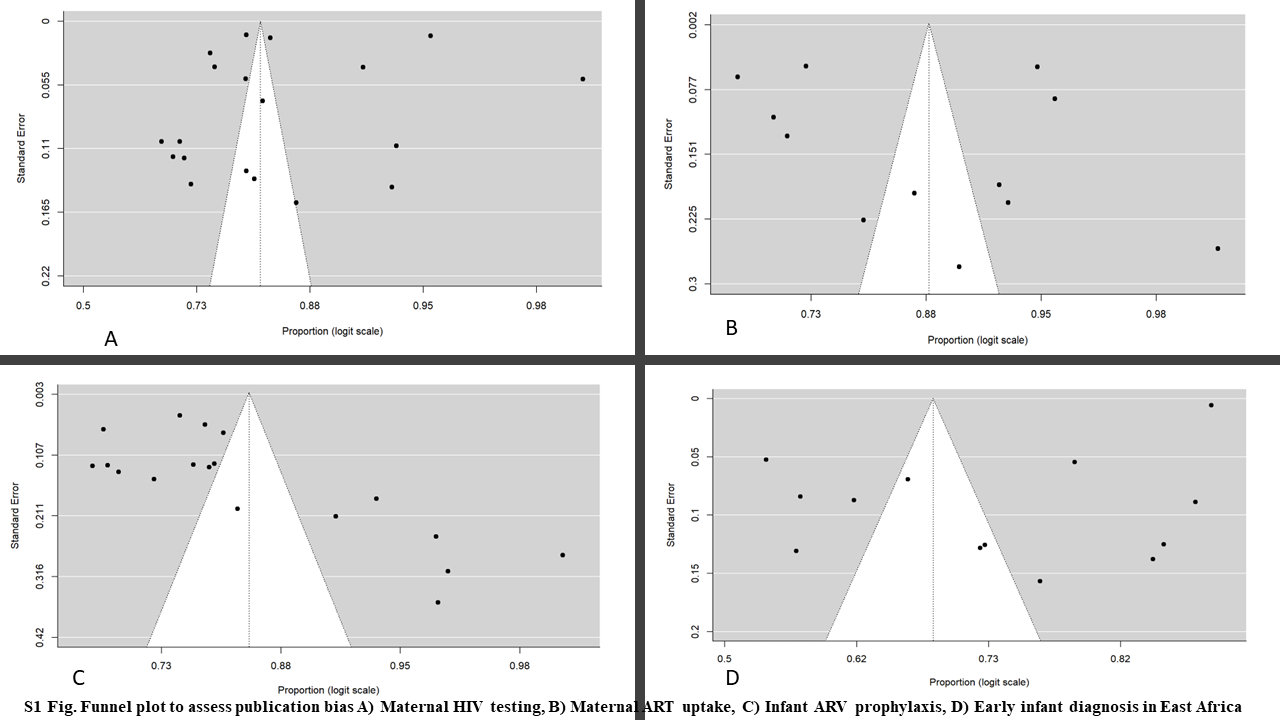

Supplement: S1 Fig — (TIF) [file pone.0300606.s001.tif]
