# Supplementary material for: Understanding the uptake and determinants of prevention of mother-to-child transmission of HIV services in East Africa: Mixed methods systematic review and meta-analysis
Source: PLoS One. 2024 Apr 18;19(4):e0300606. doi: 10.1371/journal.pone.0300606 (PMC11025786; doi:10.1371/journal.pone.0300606)
Supplement: S2 Table — (DOC) [file pone.0300606.s003.doc]

S2 Table: Search strategy

| Data base | Search | Result |
| --- | --- | --- |
| **PubMed** | ((("pregnant women"[tiab] OR "Pregnant Women"[Mesh] OR "HIV positive mothers"[tiab] OR "PMTCT mothers"[tiab] OR "Lactating mother"[tiab] OR "Breast Feeding"[Mesh] OR "breastfeeding mother"[tiab] OR "HIV exposed infants"[tiab] OR "HIV exposed child*"[tiab]) AND ("option b+"[tiab] OR "b plus"[tiab] OR "lifelong antiretroviral therapy" [tiab] OR "universal antiretroviral therapy"[tiab] OR PMTCT[tiab] OR "prevention mother-to-child transmission"[tiab] OR "prevention mother to child transmission"[tiab] OR "elimination of mother-to-child transmission"[tiab] OR "elimination of mother to child transmission"[tiab] OR "prevention of vertical Transmission"[tiab] OR "prevention of parent to child transmission"[tiab] OR "highly active Antiretroviral Therapy"[tiab] OR HAART[tiab] OR "antiretroviral therap*"[tiab] OR ART[tiab] OR "Triple Therapy"[tiab] OR ARV[tiab] OR "antiretroviral"[tiab] OR "anti-retroviral"[tiab] OR "HIV test*"[tiab] OR "opt-out HIV test*"[tiab] OR "counselling and testing"[tiab] OR VCT[tiab] OR "early infant diagnosis"[tiab] OR "infant testing"[tiab] OR "infant HIV testing"[tiab])) AND (Uptake[tiab] OR Utilization[tiab] OR Factor*[tiab] OR correlates[tiab] OR Determinant*[tiab] OR Predicator*[tiab] OR facilitator*[tiab] OR Barrier*[tiab])) AND ("Africa, Eastern" [Mesh] OR "East Africa*"[tiab] OR Burundi* [tiab] OR Djibouti* [tiab] OR Eritrea* [tiab] OR Ethiopia* [tiab] OR Kenya* [tiab] OR Mauritius* [tiab] OR Mayotte* [tiab] OR Malawi* [tiab] OR Mozambique* [tiab] OR Reunion* [tiab] OR Rwanda* [tiab] OR Somalia* [tiab] OR Sudan* [tiab] OR "South Sudan*" [tiab] OR Tanzania* [tiab] OR Uganda* [Tiab] OR Zambia* [tiab] OR Zimbabwe* [tiab] OR Madagascar* [tiab] OR Seychelles* [tiab] OR Comoros* [tiab]) | 411 |
| SCOPUS | ( TITLE-ABS-KEY ( "pregnant women" OR "HIV positive mothers" OR "PMTCT mothers" OR "Lactating mother" OR "breastfeeding mother" OR "HIV exposed infants" OR "HIV exposed child*" ) AND TITLE-ABS-KEY ( "option b+" OR "b plus" OR "lifelong antiretroviral therapy" OR "universal antiretroviral therapy" OR pmtct OR "prevention mother-to-child transmission" OR "prevention mother to child transmission" OR "elimination of mother-to-child transmission" OR "elimination of mother to child transmission" OR "prevention of vertical Transmission" OR "prevention of parent to child transmission" OR "highly active Antiretroviral Therapy" OR haart OR "antiretroviral therap*" OR art OR "Triple Therapy" OR arv OR "antiretroviral" OR "anti-retroviral" OR "HIV test*" OR "opt-out HIV test*" OR "voluntary counselling and testing" OR vct OR "early infant diagnosis" OR "infant testing" OR "infant HIV testing" ) AND TITLE-ABS-KEY ( uptake OR utilization OR factor* OR correlates OR determinant* OR predicator* OR facilitator* OR barrier* ) AND TITLE-ABS-KEY ( "East Africa*" OR burundi* OR djibouti* OR eritrea* OR ethiopia* OR kenya* OR mauritius* OR mayotte* OR malawi* OR mozambique* OR reunion* OR rwanda* OR somalia* OR sudan* OR "South Sudan*" OR tanzania* OR uganda* OR zambia* OR zimbabwe* OR madagascar* OR seychelles* OR comoros* ) ) | 615 |
| CINHAL | ( MH "Pregnant Women" OR TI "pregnant women” OR AB "pregnant women” OR TI "HIV positive mothers" OR AB "HIV positive mothers" OR TI "PMTCT mothers" OR AB "PMTCT mothers" OR TI "Lactating mother" OR AB "Lactating mother" OR MH "Breast Feeding" OR TI "breastfeeding mother" OR AB "breastfeeding mother" OR TI "HIV exposed infants" OR AB “HIV exposed infants" OR TI "HIV exposed child*" OR AB "HIV exposed child*" ) AND ( TI "option b+" OR AB "option b+" OR TI "b plus" OR AB "b plus" OR TI "lifelong antiretroviral therapy" OR AB "lifelong antiretroviral therapy" OR TI "universal antiretroviral therapy" OR AB "universal antiretroviral therapy" OR TI pmtct OR AB pmtct OR TI "prevention mother-to-child transmission" OR AB "prevention mother-to-child transmission" OR TI "prevention mother to child transmission" OR AB "prevention mother to child transmission" OR TI "elimination of mother-to-child transmission" OR AB "elimination of mother-to-child transmission" OR TI "elimination of mother to child transmission" OR AB "elimination of mother to child transmission" OR TI "prevention of vertical Transmission" OR AB "prevention of vertical Transmission" OR TI "prevention of parent to child transmission" OR AB "prevention of parent to child transmission" OR TI "highly active Antiretroviral Therapy" OR AB "highly active Antiretroviral Therapy" OR TI haart OR AB haart OR TI "antiretroviral therap*" OR AB "antiretroviral therap*" OR TI art OR AB art OR TI "Triple Therapy" OR AB "Triple Therapy" OR TI arv OR AB arv OR TI "antiretroviral" OR AB "antiretroviral" OR TI "anti-retroviral" OR AB "anti-retroviral" OR TI "HIV test*" OR AB "HIV test*" OR TI "opt-out HIV test*" OR AB "opt-out HIV test*" OR TI "voluntary counselling and testing" OR AB "voluntary counselling and testing" OR TI vct OR AB vct OR TI "early infant diagnosis" OR AB "early infant diagnosis" OR TI "infant testing" OR AB "infant testing" OR TI " infant HIV testing" OR AB "infant HIV testing" ) AND ( TI uptake OR AB uptake OR TI utilization OR AB utilization OR TI factor* OR AB factor* OR TI correlates OR AB correlates OR TI determinant* OR AB determinant* OR TI predicator* OR AB predicator* OR TI facilitator* OR AB facilitator* OR TI barrier* OR AB barrier* ) AND ( MH "Africa, Eastern" OR TI "East Africa*" OR AB "East Africa*" OR TI Burundi* OR AB Burundi* OR TI Djibouti* OR AB Djibouti* OR TI Eritrea* OR AB Eritrea* OR TI Ethiopia* OR AB Ethiopia* OR TI Kenya* OR AB Kenya* OR TI Mauritius* OR AB Mauritius* OR TI Mayotte* OR AB Mayotte* OR TI Malawi* OR AB Malawi* OR TI Mozambique* OR AB Mozambique* OR TI Reunion* OR AB Reunion* OR TI Rwanda* OR AB Rwanda* OR TI Somalia* OR AB Somalia* OR TI Sudan* OR AB Sudan* OR TI "South Sudan*" OR AB "South Sudan*" OR TI Tanzania* OR AB Tanzania* OR TI Uganda* OR AB Uganda* OR TI Zambia* OR AB Zambia* OR TI Zimbabwe* OR AB Zimbabwe* OR TI Madagascar* OR AB Madagascar* OR TI Seychelles* OR AB Seychelles* OR TI Comoros* OR AB Comoros* ) ) | 161 |
| Embase | 1. exp pregnant women/ or pregnant women.ti,ab. or HIV positive mothers.ti,ab. or PMTCT mothers.ti,ab. or Lactating mother.ti,ab. or exp Breast Feeding/ or breastfeeding mother.ti,ab. or HIV exposed infants.ti,ab. or HIV exposed child*.ti,ab. 2. (option b+ or b plus or lifelong antiretroviral therapy or universal antiretroviral therapy or PMTCT or prevention mother-to-child transmission or prevention mother to child transmission or elimination of mother-to-child transmission or elimination of mother to child transmission or prevention of vertical Transmission or prevention of parent to child transmission or highly active Antiretroviral Therapy or HAART or antiretroviral therap* or ART or Triple Therapy or ARV or antiretroviral or anti-retroviral or HIV test* or opt-out HIV test* or "voluntary counselling and testing" or VCT or early infant diagnosis or infant testing or infant HIV testing).ti,ab. 3. (Uptake or Utili?ation or Factor* or correlates or Determinant* or Predicator* or facilitator* or Barrier*).ti,ab. 4. exp Africa, Eastern/ or East Africa*.ti,ab. or Burundi*.ti,ab. or Djibouti*.ti,ab. or Eritrea*.ti,ab. or Ethiopia*.ti,ab. or Kenya*.ti,ab. or Mauritius*.ti,ab. or Mayotte*.ti,ab. or Malawi*.ti,ab. or Mozambique*.ti,ab. or Reunion*.ti,ab. or Rwanda*.ti,ab. or Somalia*.ti,ab. or Sudan*.ti,ab. or South Sudan*.ti,ab. or Tanzania*.ti,ab. or Uganda*.ti,ab. or Zambia*.ti,ab. or Zimbabwe*.ti,ab. or Madagascar*.ti,ab. or Seychelles*.ti,ab. or Comoros*.ti,ab. 5. 1 and 2 and 3 and 4 6. limit 5 to (human and english and yr="2012 - 2022") | **1023** |
| Global Index Medicus (GIM), | (tw:((pregnant women) OR (HIV positive mothers) OR (PMTCT mothers) OR (Lactating mother) OR (breastfeeding mother) OR (HIV exposed infants) OR (HIV exposed child*) )) AND (tw:((option b+) OR (b plus) OR (lifelong antiretroviral therapy) OR (universal antiretroviral therapy) OR (pmtct ) OR (prevention mother-to-child transmission) OR (prevention mother to child transmission) OR (elimination of mother-to-child transmission) OR (elimination of mother to child transmission) OR (prevention of vertical Transmission) OR (prevention of parent to child transmission) OR (highly active Antiretroviral Therapy) OR (haart) OR (antiretroviral therap*) OR (art) OR (Triple Therapy) OR (arv) OR (antiretroviral) OR (anti-retroviral) OR (HIV test*) OR (opt-out HIV test*) OR (voluntary counselling and testing) OR (vct) OR (early infant diagnosis) OR (infant testing) OR (infant HIV testing ) )) AND (tw:((uptake) OR (utilization) OR (factor*) OR (correlates) OR (determinant*) OR (predicator*) OR (facilitator*) OR (barrier*) )) AND (tw:(("East Africa*") OR (burundi*) OR (djibouti*) OR (eritrea*) OR (ethiopia*) OR (kenya*) OR (mauritius*) OR (mayotte*) OR (malawi*) OR (mozambique*) OR (reunion*) OR (rwanda*) OR (somalia*) OR (sudan*) OR (South Sudan*) OR (tanzania*) OR (uganda*) OR (zambia*) OR (zimbabwe*) OR (madagascar*) OR (seychelles*) OR (comoros*))) | **14** |
|  | TOTAL | **2224** |

Date limiter: Date: 01/01/2012; language: English

Date of search: March 30/05/2022
