# Supplementary material for: Understanding the uptake and determinants of prevention of mother-to-child transmission of HIV services in East Africa: Mixed methods systematic review and meta-analysis
Source: PLoS One. 2024 Apr 18;19(4):e0300606. doi: 10.1371/journal.pone.0300606 (PMC11025786; doi:10.1371/journal.pone.0300606)
Supplement: S3 Table — (DOCX) [file pone.0300606.s004.docx]

**S3 Table:** Methodological quality assessment from the Mixed Methods Appraisal Tool (MMAT)

|  | **1** | **2** | **3** | **4** | **5** | **6** | **7** | **The overall quality of the study** |
| --- | --- | --- | --- | --- | --- | --- | --- | --- |
| **Quantitative study** |  |  |  |  |  |  |  |  |
| Abtew 2015 (24) | Y | Y | Y | Y | Y | Y | Y | 7/7 (high) |
| Ahoua 2020 (49) | Y | Y | Y | Y | Y | Y | Y | 7/7 (high) |
| Akal 2018 (25) | Y | Y | Y | N | Y | Y | Y | 6/7 (high) |
| Alemu 2017 (26) | Y | Y | Y | Y | Y | Y | Y | 7/7 (high) |
| Astawesegn 2021 (23) | Y | Y | Y | Y | Y | Y | Y | 7/7(high) |
| Augustine 2021 (64) | Y | Y | Y | Y | Y | Y | N | 6/7(high) |
| Berhan 2014 (27) | Y | Y | Y | Y | Y | Y | Y | 7/7(high) |
| Bwana 2018 (53) | Y | Y | Y | Y | Y | Y | Y | 7/7(high) |
| Desta 2019(28) | Y | Y | Y | Y | Y | Y | Y | 7/7(high) |
| Dzangare et al 2016(65) | Y | Y | N | Y | Y | Y | Y | 6/7(high) |
| Ebuy et al 2020(29) | Y | Y | Y | Y | Y | Y | Y | 7/7(high) |
| Ejigu et al 2018(30) | Y | Y | Y | Y | Y | Y | Y | 7/7(high) |
| Gaitho et al 2021(41) | Y | Y | Y | Y | Y | Y | Y | 7/7(high) |
| Gamell et al 2017(54) | Y | Y | Y | Y | N | U | Y | 5/7(moderate) |
| Gebeyehu et al 2019(31) | Y | Y | Y | Y | Y | Y | Y | 7/7(high) |
| Gebremedhin et al 2018(32) | Y | Y | N | Y | Y | Y | Y | 6/7(high) |
| Gebresillassie et al 2019(33) | Y | Y | Y | Y | Y | Y | Y | 7/7(high) |
| Haider et al 2022(42) | Y | Y | Y | Y | Y | Y | Y | 7/7(high) |
| Hampanda et al 2017(62) | Y | Y | Y | Y | Y | Y | Y | 7/7(high) |
| Kebede et al 2014(34) | Y | Y | N | N | Y | Y | Y | 5/7(moderate) |
| Lain et al 2020 (51) | Y | Y | Y | Y | Y | U | Y | 6/7(high) |
| Makau et al 2015 (43) | Y | Y | Y | Y | N | Y | Y | 6/7(high) |
| Moges et al 2017 (35) | Y | Y | Y | Y | Y | Y | Y | 7/7(high) |
| Ng'ambi et al 2022 (47) | Y | Y | Y | N | Y | Y | Y | 6/7(high) |
| Nungu et al 2019 (56) | Y | Y | Y | Y | Y | Y | Y | 7/7(high) |
| Ongaki et al 2019 (44) | Y | Y | N | N | N | Y | Y | 4/7(moderate) |
| Tadewos et al 2020 (36) | Y | Y | Y | Y | Y | Y | Y | 7/7(high) |
| Thidor et al 2019 (52) | Y | Y | U | N | Y | U | Y | 4/7(moderate) |
| Tsehay et al 2019 (37) | Y | Y | Y | Y | N | Y | Y | 6/7(high) |
| Van Lettow et al 2018 (48) | Y | Y | Y | Y | Y | Y |  | 7/7(high) |
| Wanyenze et al 2018 (66) | Y | Y | N | Y | Y | Y | Y | 6/7(high) |
| Workagegn et al 2015 (38) | Y | Y | Y | Y | Y | Y | Y | 7/7(high) |
| Wudineh et al 2016 (39) | Y | Y | Y | Y | Y | Y | Y | 7/7(high) |
| Zegeye 2020 (40) | Y | Y | Y | Y | Y | Y | Y | 7/7(high) |
| Semali et al 2014 (68) | Y | Y | Y | Y | Y | Y | Y | 7/7(high) |
| Deressa et al 2014 (69) | Y | Y | Y | Y | Y | Y | Y | 7/7(high) |
| Ford et al 2018 (70) | Y | Y | Y | N | Y | Y | Y | 6/7(high) |
| Olana et al 2016 (71) | Y | Y | Y | Y | Y | Y | Y | 7/7(high) |
| Yaya et al 2019 (72) | Y | Y | Y | Y | Y | Y | Y | 7/7(high) |
| McCoy et al 2015 (73) | Y | Y | Y | Y | Y | Y | Y | 7/7(high) |
| **Qualitative study** |  |  |  |  |  |  |  |  |
| Bobrow 2016 (45) | Y | Y | Y | Y | Y | Y | Y | 7/7(high) |
| Buleza Lamucene 2022 (50) | Y | Y | Y | N | Y | Y | Y | 6/7(high) |
| Buregyeya 2017 (59) | Y | Y | Y | Y | Y | Y | Y | 7/7(high) |
| Cataldo 2017(46) | Y | Y | Y | Y | Y | Y | Y | 7/7(high) |
| Kanguya et al 2022(63) | Y | Y | Y | Y | Y | Y | Y | 7/7(high) |
| Oshosen et al 2021 (57) | Y | Y | Y | Y | Y | Y | Y | 7/7(high) |
| Chadambuka et al 2018 (67) | Y | Y | Y | Y | Y | Y | Y | 7/7(high) |
| Kim et al 2016 (74) | Y | Y | Y | Y | Y | Y | Y | 7/7(high) |
| **Mixed method** |  |  |  |  |  |  |  |  |
| Bergmann 2017 (58) | Y | Y | Y | Y | Y | Y | Y | 7/7(high) |
| Konje et al 2018 (55) | Y | Y | Y | Y | N | N | Y | 5/7 (moderate) |
| Mukose et al 2021 (60) | Y | Y | Y | Y | Y | Y | Y | 7/7(high) |
| Mustapha et al 2018 (61) | Y | Y | Y | Y | Y | Y | Y | 7/7(high) |

**Screening questions for all types of studies:** *(1) Are there clear research questions? (2) Do the collected data allow us to address the research questions?*

Q**uantitative studies: *i) Descriptive*** *q****uantitative studies:*** *(3) Is the sampling strategy relevant to address the research question? (4) Is the sample representative of the target population? (5) Are the measurements appropriate? (6) Is the risk of nonresponse bias low? (7) Is the statistical analysis appropriate to answer the research question?* ***ii) Analytical*** *q****uantitative studies*** *(3) Are the participants representative of the target population? (4) Are measurements appropriate regarding both the outcome and intervention (or exposure)? (5) Are there complete outcome data? (6) Are the confounders accounted for in the design and analysis? (7) During the study period, is the intervention administered (or exposure occurred) as intended?*

**Qualitative studies**: *(3) Is the qualitative approach appropriate to answer the research question? (4) Are the qualitative data collection methods adequate to address the research question? (5) Are the findings adequately derived from the data? (6) Is the interpretation of results sufficiently substantiated by data? (7) Is there coherence between qualitative data sources, collection, analysis, and interpretation?*

**Mixed methods studies:** *(3)* *Is there an adequate rationale for using a mixed methods design to address the research question? (4) Are the different components of the study effectively integrated to answer the research question? (5) Are the outputs of the integration of qualitative and quantitative components adequately interpreted? (6) Are divergences and inconsistencies between quantitative and qualitative results adequately addressed? (7) Do the different components of the study adhere to the quality criteria of each tradition of the methods involved?*

Each item was rated “Y = Yes”, “N = No”, or “U = Unclear” was awarded where not enough information was provided. High quality: meets ≥ 7 criteria, Moderate quality: meets ≥ 4 criteria, Low quality: < 4 criteria.
