# Supplementary material for: Understanding the uptake and determinants of prevention of mother-to-child transmission of HIV services in East Africa: Mixed methods systematic review and meta-analysis
Source: PLoS One. 2024 Apr 18;19(4):e0300606. doi: 10.1371/journal.pone.0300606 (PMC11025786; doi:10.1371/journal.pone.0300606)
Supplement: S4 Table — (DOCX) [file pone.0300606.s005.docx]

**S4 Table: The pooled proportion of PMTCT cascades, 95% CI and heterogeneity estimate with a P-value for the subgroup analysis.**

| **Cascades** | **Subgroup variables** | **No of studies** | **Proportion (%) [95CI]** | **Weight** | **I^2^ (P-value)** |
| --- | --- | --- | --- | --- | --- |
| Maternal HIV testing | | | | | |
|  | **Country** | | | | |
|  | Ethiopia | 9 | 75.48[58.53; 87.04] | 40.8 | 99% (p < 0.01) |
|  | Kenya | 1 | 83.90[83.52; 84.27] | 4.6 |  |
|  | Malawi | 1 | 98.80[98.68; 98.91] | 4.6 |  |
|  | Mozambique | 2 | 89.02[54.65; 98.20] | 9.2 | 100% (p = 0) |
|  | Multiple countries* | 1 | 80.80[80.44; 81.15] | 4.6 |  |
|  | South Sudan | 1 | 72.11[66.24; 77.31] | 4.5 |  |
|  | Tanzania | 4 | 76.27[51.37; 90.72] | 18.3 | 100% (p< 0.01) |
|  | Uganda | 1 | 95.69[93.27; 97.27] | 4.4 |  |
|  | Zimbabwe | 2 | 87.55[71.79; 95.11] | 9.2 | 100% (p< 0.01) |
|  | Overall | 22 | 82.69[75.62; 88.03] | 100.0 | 100% (p = 0) |
|  | **Year of Publication** | | | | |
|  | 2012-2014 | 2 | 87.38[59.94; 96.97] | 9.0 | 99% (p < 0.01) |
|  | 2015-2017 | 5 | 85.32[74.55; 92.02] | 22.8 | 99% (p < 0.01) |
|  | 2018-2020 | 13 | 80.80[62.51; 91.39] | 59.0 | 100% (p = 0) |
|  | 2021-2022 | 2 | 82.40[79.16; 85.24] | 9.2 | 99% (p < 0.01) |
|  | Overall | 22 | 82.69[75.62; 88.03] | 100.0 | 100% (p = 0) |
|  | **Study setting** | | | | |
|  | Facility | 15 | 84.68[72.87; 91.91] | 67.9 | 100% (p = 0) |
|  | Community | 7 | 77.85[69.69; 84.31] | 32.1 | 100% (*p* = 0) |
|  | Overall | 22 | 82.69[75.62; 88.03] | 100.0 | 100% (p = 0) |
| **Maternal ART uptake** | | | | | |
|  | **Country** | | | | |
|  | Ethiopia | 2 | 84.37[42.24; 97.55] | 16.7 | 99% (p < 0.01) |
|  | Kenya | 2 | 80.34[61.91; 91.13] | 16.8 | 95% (p < 0.01) |
|  | Malawi | 1 | 95.76[95.01; 96.40] | 8.4 |  |
|  | Mozambique | 2 | 97.66[89.95; 99.49] | 16.6 | 97% (*p* < 0.01) |
|  | Uganda | 2 | 88.62[71.07; 96.11] | 16.5 | 94% (p < 0.01) |
|  | Zambia | 1 | 68.84[63.13; 74.03] | 8.4 |  |
|  | Zimbabwe | 2 | 78.69[35.89; 96.05] | 16.6 | 98% (p < 0.01) |
|  | Overall | 12 | 88.33[78.59; 93.98] | 100 | 99% (*p* = 0) |
|  | **Year of Publication** | | | | |
|  | 2012-2014 | 1 | 93.78[90.96; 95.76] | 8.3 |  |
|  | 2015-2017 | 3 | 73.70[59.20; 84.41] | 25.0 | 96% (p < 0.01) |
|  | 2018-2020 | 6 | 92.08[83.97; 96.27] | 50.0 | 99% (p < 0.01) |
|  | 2021-2022 | 2 | 85.64[53.51; 96.86] | 16.8 | 99% (p < 0.01) |
|  | Overall | 12 | 88.33[78.59; 93.98] | 100 | 99% (*p* = 0) |
|  | **Study setting** | | | | |
|  | Facility | 10 | 91.03[82.57; 95.60] | 83.1 | 99% (p < 0.01) |
|  | Community | 2 | 63.71[53.56; 72.76] | 16.9 | 89% (p < 0.01) |
|  | Overall | 12 | 88.33[78.59; 93.98] | 100 | 99% (*p* = 0) |
| Infant ARV prophylaxis | | | | | |
|  | **Country** | | | | |
|  | Ethiopia | 8 | 90.66[82.22; 95.32] | 42.8 | 97% (p < 0.01) |
|  | Kenya | 3 | 72.99[62.73; 81.27] | 17.4 | 93% (p < 0.01) |
|  | Malawi | 1 | 76.04[74.61; 77.43] | 6.0 |  |
|  | Mozambique | 1 | 82.02[79.93; 83.94] | 5.9 |  |
|  | Tanzania | 1 | 83.70[77.64; 88.36] | 5.4 |  |
|  | Uganda | 1 | 80.21[75.92; 83.89] | 5.8 |  |
|  | Zambia | 1 | 63.41[57.56; 68.88] | 5.8 |  |
|  | Zimbabwe | 2 | 91.88[20.76; 99.80] | 10.9 | 99% (p < 0.01) |
|  | Overall |  | 84.98[80.78; 88.39] | 100.0 | 97% (p < 0.01) |
|  | **Year of Publication** | | | | |
|  | 2012-2014 | 2 | 71.78[48.42; 87.33] | 11.6 | 97% (p < 0.01) |
|  | 2015-2017 | 6 | 78.60[68.64; 86.03] | 34.1 | 96% (p < 0.01) |
|  | 2018-2020 | 8 | 88.58[82.52; 92.72] | 43.4 | 97% (p < 0.01) |
|  | 2021-2022 | 2 | 94.53[47.90; 99.69] | 11.0 | 99% (p < 0.01) |
|  | Overall |  | 84.98[80.78; 88.39] | 100.0 | 97% (p < 0.01) |
|  | **Study setting** | | | | |
|  | Facility | 16 | 86.83[82.98; 89.92] | 88.3 | 97% (p < 0.01) |
|  | Community | 2 | 62.76[60.14; 65.30] | 11.7 | 0% (p = 0.80) |
|  | Overall | 18 | 84.98[80.78; 88.39] | 100.0 | 97% (p < 0.01) |
| Early infant diagnosis | | | | | |
|  | **Country** | | | | |
|  | Ethiopia | 8 | 65.60[51.03; 77.73] | 53.1 | 98% (p < 0.01) |
|  | Kenya | 2 | 69.10[44.30; 86.28] | 13.4 | 98% (p < 0.01) |
|  | Malawi | 2 | 73.12[34.28; 93.42] | 13.5 | 100% (p < 0.01 |
|  | Tanzania | 1 | 57.12[53.04; 61.10] | 6.7 |  |
|  | Zambia | 1 | 72.81[67.67; 77.41] | 6.6 |  |
|  | Zimbabwe | 1 | 85.60[83.32; 87.62] | 6.7 |  |
|  | Overall | 15 | 68.77[57.63; 78.09] | 100 | 100% (p = 0) |
|  | **By the year of Publication** | | | | |
|  | 2012-2014 | 2 | 32.63[19.37; 49.40] | 13.3 | 95% (p < 0.01) |
|  | 2015-2017 | 5 | 71.17[62.84; 78.27] | 33.2 | 93% (p < 0.01) |
|  | 2018-2020 | 5 | 67.84[56.56; 77.37] | 33.3 | 97% (p < 0.01) |
|  | 2021-2022 | 3 | 83.89[78.49; 88.14] | 20.2 | 98% (p < 0.01) |
|  | Overall | 15 | 68.77[57.63; 78.09] | 100 | 100% (p = 0) |
|  | **By study setting** | | | | |
|  | Facility | 15 | 68.77[57.63; 78.09] | 100 | 100% (p = 0) |
|  | Community | NA | NA | NA | NA |
|  | Overall | 15 | 68.77[57.63; 78.09] | 100 | 100% (p = 0) |

*NA: Not available; *Burundi, Comoros, Ethiopia, Kenya, Malawi, Mozambique, Rwanda, Uganda, Zambia, and Zimbabwe.*
