# Supplementary material for: Understanding the uptake and determinants of prevention of mother-to-child transmission of HIV services in East Africa: Mixed methods systematic review and meta-analysis
Source: PLoS One. 2024 Apr 18;19(4):e0300606. doi: 10.1371/journal.pone.0300606 (PMC11025786; doi:10.1371/journal.pone.0300606)
Supplement: Attachment — Submitted filename: PONE-D-23-04697_BN_reviewers comments_15thJuly2023.pdf [file pone.0300606.s006.pdf]

PLOS ONE

Understanding the uptake and determinants of prevention of mother-to-child transmission of HIV services in East Africa: Mixed methods systematic review and meta-analysis

--Manuscript Draft--

Manuscript Number:	PONE-D-23-04697
Article Type:	Research Article
Full Title:	Understanding the uptake and determinants of prevention of mother-to-child transmission of HIV services in East Africa: Mixed methods systematic review and meta-analysis
Short Title:	Prevention of mother-to-child transmission of HIV service in East Africa: Systematic review and meta analysis
Corresponding Author:	Feleke Hailemichael Astawesegn, MPH<br>Hawassa University College of Medicine and Health Sciences<br>Hawassa, ETHIOPIA
Keywords:	PMTCT, HIV diagnosis, HIV prophylaxis, Factors, East Africa
Abstract:	Background : Prevention of mother-to-child transmission (PMTCT) of HIV service is conceptualized as a series of cascades. A low rate of cascade completion by mothers results in an increased risk of HIV transmission to their infants. Therefore, this review aimed to understand the uptake and determinants of key PMTCT of HIV services cascades in East Africa<br>Methods: We searched CINAHL, EMBASE, MEDLINE, Scopus, and AIM databases using a predetermined search strategy to identify studies published from January 2012 through to March 2022 on the uptake and determinants of PMTCT of HIV services. The quality of the included studies was assessed using the Mixed Methods Appraisal Tool. A random-effects model was used to obtain pooled estimates of (i) maternal HIV testing (ii) maternal ART initiation, (iii) infant ARV prophylaxis and (iv) early infant diagnosis (EID). Factors from quantitative studies were reviewed using a coding template based on the domains of the Andersen model (i.e. environmental, predisposing, enabling and need factors) and qualitative studies were reviewed using a thematic synthesis approach.<br>Results: The searches yielded 2231 articles and we systematically reduced to 52 included studies. Forty quantitative, eight qualitative, and four mixed methods papers were located containing evidence on the uptake and determinants of PMTCT services. The pooled proportions of maternal HIV test and ART uptake in East Africa were 82.7% (95% CI: 75.6– 88%) and 88% (95% CI: 78.6–94%). Similarly, the pooled estimates of infant ARV prophylaxis and EID uptake were 85% (95% CI: 80.8-88.4%) and 68.8% (95% CI: 57.6-78.1) respectively. Key factors included place of residence, stigma, women's age, maternal and paternal education, marital status, socioeconomic status (SES), knowledge about HIV/PMTCT/MTCT, access to a health facility, attitudes/perception towards provider-initiated HIV testing and counselling (PITC), previous use of maternal services, and health care factors (for example, shortage of resources and lack of follow-up supervision).<br>Conclusion: Most of the identified factors were modifiable which should be considered in policymaking and planning of interventions. Hence, promoting education, and economic empowerment, strengthening staff supervision, improving access and integration with maternal and child health services, and reducing stigma with active community participation are recommended.
Order of Authors:	Feleke Hailemichael Astawesegn, MPH
	Haider Mannan, PhD
	Virginia Stulz, PhD
	Elizabeth Conroy, PhD
Additional Information:	
Question	Response

Powered by Editorial Manager® and ProduXion Manager® from Aries Systems Corporation

**Financial Disclosure**<br><br>Enter a financial disclosure statement that describes the sources of funding for the work included in this submission. Review the submission guidelines for detailed requirements. View published research articles from *PLOS ONE* for specific examples.<br><br>This statement is required for submission and **will appear in the published article** if the submission is accepted. Please make sure it is accurate.<br><br>**Unfunded studies**<br>Enter: *The author(s) received no specific funding for this work.*<br><br>**Funded studies**<br>Enter a statement with the following details:<br>• Initials of the authors who received each award<br>• Grant numbers awarded to each author<br>• The full name of each funder<br>• URL of each funder website<br>• Did the sponsors or funders play any role in the study design, data collection and analysis, decision to publish, or preparation of the manuscript?<br>• **NO** - Include this sentence at the end of your statement: *The funders had no role in study design, data collection and analysis, decision to publish, or preparation of the manuscript.*<br>• **YES** - Specify the role(s) played.<br><br>* typeset	The author(s) received no specific funding for this work.
**Competing Interests**<br><br>Use the instructions below to enter a competing interest statement for this submission. On behalf of all authors, disclose any competing interests that could be perceived to bias this work—acknowledging all financial support and any other relevant financial or non-financial competing interests.<br><br>This statement is required for submission	The authors have declared that no competing interests exist.

and **will appear in the published article** if the submission is accepted. Please make sure it is accurate and that any funding sources listed in your Funding Information later in the submission form are also declared in your Financial Disclosure statement.<br><br>View published research articles from *PLOS ONE* for specific examples.<br><br>**NO authors have competing interests**<br><br>Enter: *The authors have declared that no competing interests exist.*<br><br>**Authors with competing interests**<br><br>Enter competing interest details beginning with this statement:<br><br>*I have read the journal's policy and the authors of this manuscript have the following competing interests: [insert competing interests here]*<br><br>* typeset	
**Ethics Statement**<br><br>Enter an ethics statement for this submission. This statement is required if the study involved:<br><br>• Human participants<br>• Human specimens or tissue<br>• Vertebrate animals or cephalopods<br>• Vertebrate embryos or tissues<br>• Field research<br><br>Write "N/A" if the submission does not require an ethics statement.<br><br>General guidance is provided below. Consult the submission guidelines for detailed instructions. **Make sure that all information entered here is included in the Methods section of the manuscript.**	N/A

**Format for specific study types**<br><br>**Human Subject Research (involving human participants and/or tissue)**<br>• Give the name of the institutional review board or ethics committee that approved the study<br>• Include the approval number and/or a statement indicating approval of this research<br>• Indicate the form of consent obtained (written/oral) or the reason that consent was not obtained (e.g. the data were analyzed anonymously)<br><br>**Animal Research (involving vertebrate animals, embryos or tissues)**<br>• Provide the name of the Institutional Animal Care and Use Committee (IACUC) or other relevant ethics board that reviewed the study protocol, and indicate whether they approved this research or granted a formal waiver of ethical approval<br>• Include an approval number if one was obtained<br>• If the study involved *non-human primates*, add *additional details* about animal welfare and steps taken to ameliorate suffering<br>• If anesthesia, euthanasia, or any kind of animal sacrifice is part of the study, include briefly which substances and/or methods were applied<br><br>**Field Research**<br><br>Include the following details if this study involves the collection of plant, animal, or other materials from a natural setting:<br>• Field permit number<br>• Name of the institution or relevant body that granted permission	
**Data Availability**<br><br>Authors are required to make all data underlying the findings described fully available, without restriction, and from the time of publication. PLOS allows rare exceptions to address legal and ethical concerns. See the PLOS Data Policy and FAQ for detailed information.	Yes - all data are fully available without restriction

A Data Availability Statement describing where the data can be found is required at submission. Your answers to this question constitute the Data Availability Statement and **will be published in the article**, if accepted.<br><br>**Important:** Stating 'data available on request from the author' is not sufficient. If your data are only available upon request, select 'No' for the first question and explain your exceptional situation in the text box.<br><br>Do the authors confirm that all data underlying the findings described in their manuscript are fully available without restriction?	
**Describe where the data may be found in full sentences. If you are copying our sample text, replace any instances of XXX with the appropriate details.**<br><br>• If the data are **held or will be held in a public repository**, include URLs, accession numbers or DOIs. If this information will only be available after acceptance, indicate this by ticking the box below. For example: *All XXX files are available from the XXX database (accession number(s) XXX, XXX.).*<br>• If the data are all contained **within the manuscript and/or Supporting Information files**, enter the following: *All relevant data are within the manuscript and its Supporting Information files.*<br>• If neither of these applies but you are able to provide **details of access elsewhere**, with or without limitations, please do so. For example:<br><br>*Data cannot be shared publicly because of [XXX]. Data are available from the XXX Institutional Data Access / Ethics Committee (contact via XXX) for researchers who meet the criteria for access to confidential data.*<br><br>*The data underlying the results presented in the study are available from (include the name of the third party*	All relevant data are within the manuscript and its Supporting Information files.

and contact information or URL).<br>• This text is appropriate if the data are owned by a third party and authors do not have permission to share the data.<br><br>* typeset	
Additional data availability information:	

Manuscript Click here to access/download;Manuscript;Manuscript.docx

Understanding the uptake and determinants of prevention of mother-to-child transmission of HIV services in East Africa: Mixed methods systematic review and meta-analysis

Authors
Feleke Hailemichael Astawesegn[1,2*], Haider Mannan[1], Virginia Stulz[3], Elizabeth Conroy[1]

[1] Translational Health Research Institute (THRI), Western Sydney University, Campbelltown campus, Penrith, New South Wales 2751, Australia.

EC: e.conroy@westernsydney.edu.au HM: h.mannan@westernsydney.edu.au

[2] School of Public Health, College of Medicine and Health Sciences, Hawassa University, Ethiopia.

[3]School of Nursing and Midwifery Centre for Nursing and Midwifery Research, Western Sydney University, Nepean Hospital 1st level Court Building, Derby St, Kingswood, NSW 2340 Australia

VS: v.stulz@westernsydney.edu.au

*Corresponding Author: Feleke Hailemichael Astawesegn
Email: felekeh86@gmail.com

Abstract

Background: Prevention of mother-to-child transmission (PMTCT) of HIV service is conceptualized as a series of cascades. A low rate of cascade completion by mothers results in an increased risk of HIV transmission to their infants. Therefore, this review aimed to understand the uptake and determinants of key PMTCT of HIV services cascades in East Africa

Methods: We searched CINAHL, EMBASE, MEDLINE, Scopus, and AIM databases using a predetermined search strategy to identify studies published from January 2012 through to March 2022 on the uptake and determinants of PMTCT of HIV services. The quality of the included studies was assessed using the Mixed Methods Appraisal Tool. A random-effects model was used to obtain pooled estimates of (i) maternal HIV testing (ii) maternal ART initiation, (iii) infant ARV prophylaxis and (iv) early infant diagnosis (EID). Factors from quantitative studies were reviewed using a coding template based on the domains of the Andersen model (i.e. environmental, predisposing, enabling and need factors) and qualitative studies were reviewed using a thematic synthesis approach.

Results: The searches yielded 2231 articles and we systematically reduced to 52 included studies. Forty quantitative, eight qualitative, and four mixed methods papers were located containing evidence on the uptake and determinants of PMTCT services. The pooled proportions of maternal HIV test and ART uptake in East Africa were 82.7% (95% CI: 75.6–88%) and 88% (95% CI: 78.6–94%). Similarly, the pooled estimates of infant ARV prophylaxis and EID uptake were 85% (95% CI: 80.8-88.4%) and 68.8% (95% CI: 57.6-78.1) respectively. Key factors included place of residence, stigma, women's age, maternal and paternal education, marital status, socioeconomic status (SES), knowledge about HIV/PMTCT/MTCT, access to a health facility, attitudes/perception towards provider-initiated HIV testing and counselling (PITC), previous use of maternal services, and health care factors (for example, shortage of resources and lack of follow-up supervision).

Conclusion: Most of the identified factors were modifiable which should be considered in policymaking and planning of interventions. Hence, promoting education, and economic empowerment, strengthening staff supervision, improving access and integration with maternal and child health services, and reducing stigma with active community participation are recommended.

Keywords

HIV diagnosis, HIV prophylaxis, PMTCT, Factors, East Africa

Background

Prevention of mother-to-child transmission of HIV (PMTCT) services are an essential component of the global effort to decrease the incidence of new human immunodeficiency virus (HIV) infections and to ensure an HIV- and acquired immunodeficiency syndrome (AIDS)-free generation. For the last two decades, the implementation of the PMTCT program has substantially decreased the number of HIV-infected babies born to HIV-positive mothers worldwide through improved HIV diagnosis, care, and treatment services (1). Along with technical and financial support from international health organizations including the world health organization (2) and integration of maternal, newborn and child health services (3, 4).

Despite considerable progress in the PMTCT program, HIV remains a disease of public health importance in sub-Saharan Africa (SSA) (5) where more than two-thirds of the world's HIV-infected children live (6). The current global efforts in the fight against human immunodeficiency virus (HIV) have been focused on the virtual elimination of child HIV infection for resource-limited settings with targets of MTCT rate < 5% in breastfeeding countries and < 2% in non-breastfeeding countries (7-10). Hence, World Health Organization endorsed lifelong antiretroviral therapy for pregnant and breastfeeding women diagnosed with HIV infection and provision of nevirapine to all HIV-exposed infants for 4-6 weeks (Option B+ approach) to prevent mother-to-child HIV transmission (11).

Effectively implemented PMTCT service can reduce the risk of vertical HIV transmission from 15-45% to less than 1 % (12-14). If PMTCT programs are going to be effective, HIV-infected pregnant women must be able to navigate through complex and sequential steps called the PMTCT cascade. It refers to the sequence of steps a mother with HIV takes from diagnosis through receiving appropriate care/treatment for themselves and their newborns (15-17). It begins with all pregnant women and ends with exposed infants' HIV testing (17), which includes (but is not limited to) antenatal HIV testing, initiating antiretroviral therapy (ART) treatment during pregnancy, birth, and breastfeeding, ARV prophylaxis for HIV-exposed infants (HEI) and early infant HIV diagnosis (EID)/testing of infants at six weeks (18). Thus, successful navigation of pregnant women along the PMTCT cascade is crucial and at each step, 95% uptake is required to effectively reduce MTCT of HIV and virtually eliminate MTCT by 2030 (5, 11).

However, mother-to-child transmission (MTCT) of HIV has remained a challenge because of the cumulative dropout rate of women and their infants at each step along these cascades (19-21). A review done in SSA showed that 94% of pregnant women were tested for HIV, 70% of

those who were HIV-positive initiated ARV/ART, and 64% of the HEIs were tested for HIV at six weeks/ had an early diagnosis (22). health systems are challenged to support women's transfer along these various stages of care resulting in cumulative losses of pregnant women from the PMTCT program, with an increased risk of HIV transmission to their infants (23).

Studies worldwide have reported factors that have an association with maternal HIV test and ART services uptake, including place of residence (24-26), education level (24, 26-28), maternal age (26, 27), knowledge of HIV/AIDS and PMTCT (27, 29, 30), SES (24, 26, 31, 32), lack of privacy and confidentiality (33), women's decision-making capacity (29, 34, 35), prenatal care (26, 31), fear of disclosure and stigma (30, 36-39). Likewise, factors such as distance from a health facility (40), partner/family support (39), denial of HIV status (41), shortages of resources (41, 42), lack of knowledge (43), feelings of guilt (43), children of known HIV positive fathers (44) and maternal receipt of ART/HAART (40, 43, 45) have also been reported to affect infant ARV prophylaxis and EID services uptake.

In East Africa, the uptake and determinants of key PMTCT services cascades have not been collectively and systematically analysed and remain poorly understood. Therefore, this mixed-methods systematic review and meta-analysis aims to understand the uptake and determinants of (i) maternal HIV testing among pregnant/postpartum women, (ii) ART initiation among HIV-infected pregnant/postpartum women, (iii) initiation of ARV prophylaxis for HEIs, and (iv) EID/HIV test at six weeks of age/ in East Africa. It is hoped that providing such vital information would help policymakers design better strategies and implement targeted interventions in East Africa.

Methods

This mixed-method systematic review and meta-analysis was conducted using the Preferred Reporting Items for Systematic Reviews and Meta-analyses (PRISMA) guidelines (46) **[S1 Table].**

Inclusion criteria

This review has considered quantitative, qualitative, and mixed methods studies. Studies with a quantitative study design (such as prospective cohort, retrospective cohort, case-control, or cross-sectional study) and a qualitative design (such as phenomenology, and grounded theory) were considered in the study. For the quantitative component of the review, the exposures of interest were factors that were associated with the PMTCT cascade uptake. An exposure factor was identified when a study reported a statistically significant association between the exposure (independent) and the outcome (dependent) variable. In the qualitative component of the review, our interests were mothers' and providers' experiences and/or perceptions of the factors that affect PMTCT cascade uptake. Only studies conducted in East African countries (Burundi, Djibouti, Eritrea, Ethiopia, Kenya, Mauritius, Mayotte, Malawi, Mozambique, Réunion, Rwanda, Somalia, Sudan, South Sudan, United Republic of Tanzania, Uganda, Zambia, Zimbabwe, Madagascar, Seychelles, and Comoros) were taken into consideration (47). Studies involving pregnant, postnatal, and breastfeeding mothers and/or providers of PMTCT services from East Africa were considered. Only full-text available studies, published in English, and published in a peer-reviewed journal from 01/01/2012 to 30/05/2022 were considered (this reflects the period after which option B+ strategy was introduced in East Africa by Malawi, the first country to do so(11, 48)).

Information sources and Search strategies

Searches were performed in five databases PubMed, Scopus, EMBASE, African Index Medicus (AIM), and CINAHL. The search strategy used four search concepts including the following keywords: **Search #1:** “pregnant women", “HIV positive mother”, "PMTCT mother", "Lactating mother", "Breast Feeding", "breastfeeding mother", "HIV exposed infant", "HIV exposed child”. **Search #2:** "option b+", "b plus", "lifelong antiretroviral therapy", "universal antiretroviral therapy", PMTCT, "prevention mother-to-child transmission", "prevention mother to child transmission", "elimination of mother-to-child transmission", "elimination of mother to child transmission", "prevention of vertical Transmission", "prevention of parent to child transmission", "highly active Antiretroviral Therapy", HAART, "antiretroviral therap*", ART, "Triple Therapy", ARV, "antiretroviral", "anti-retroviral", "HIV test*", "opt-out HIV test*", "counselling and testing", VCT, "early infant diagnosis", "infant

testing", "infant HIV testing". **Search #3:** uptake, utilization, factor*, correlates, determinant*, predicator*, facilitator*, barrier*. **Search #4:** "East Africa*", Burundi*, Djibouti*, Eritrea*, Ethiopia*, Ghana*, Kenya*, Mauritius*, Mayotte*, Malawi*, Mozambique*, Reunion*, Rwanda*, Somalia*, Sudan*, "South Sudan*", Tanzania*, Uganda*, Zambia*, Zimbabwe*, Madagascar*, Seychelles*, Comoros*. The four search concepts, their synonyms and truncations by the use of the asterisk '*' where appropriate were combined using the Boolean operators 'OR', within concepts, and 'AND' to combine concepts to develop the final search strategy **[S2 Table].**

Study selection and data extraction

All identified citations were collected and uploaded into the reference management software EndNote version X9 and duplicates were removed. Articles were further screened based on titles and abstracts followed by full-text assessment by two independent reviewers (F.H and T.Y). Any disagreements that arose between the reviewers at each stage of the selection process were resolved through discussion. The results of the search and the study inclusion process were reported in the final systematic review and presented in the Preferred Reporting Items for Systematic Reviews and Meta-analyses (PRISMA) flow diagram (46). Using a structured table, the following information was extracted from each eligible article: (i) country; (ii) year of study; (iii) year of publication; (iv) study setting, (v) specific details about the participants (pregnant women, postpartum women, HEIs and PMTCT service providers); (vi) study methods/design (vii) sample size (viii) aim of the study and (ix) uptake and significant determinants or the phenomena of interest relevant to the review objective for qualitative studies.

Assessment of methodological quality

The methodological quality of studies that met the inclusion criteria was evaluated using the Mixed Methods Appraisal Tool (MMAT) version 2018 (49) that has been employed by different studies (50-52). The MMAT was developed to allow systematic reviewers to assess the methodological quality of diverse study designs, including qualitative, quantitative, and mixed methods. We used three categories to rate the quality of the studies (high, moderate, and low). Studies that score 6 or more out of 7 criteria were deemed as high; studies that scored 3 to 5 out of 7 criteria were deemed as moderate; finally, studies that scored 2 or less out of 7 criteria were considered of low quality. Quality scores for each study are presented in **[S3 Table].**

Data analysis and synthesis

Firstly, heterogeneity between included studies was checked using the I^2 test statistic and its corresponding p-value. A p-value of less than 0.05 was used to decide on the presence of heterogeneity (53). Publication bias among the studies was assessed statistically using Egger's and Begg's tests, and visually using a funnel plot. Funnel plots are scatter plots that show the effect size estimates (on the x-axis), plotted against the standard errors of the effect size (on the y-axis). The vertical line represents the estimated average effect size. Then, the pooled proportion of HIV testing, ART initiation, infant prophylaxis and EID was estimated with a 95% confidence interval (CI) using a random effect model as there was heterogeneity between studies ($p < 0.01\%$). Besides, subgroup analysis was conducted by year of publication, study settings and countries based on the random effects model for each cascade.

Secondly, factors determining PMTCT cascade uptake were reviewed according to the JBI methodology for mixed-method systematic review (MMSR) (54). This involves separate quantitative and qualitative synthesis followed by integration of the two forms of evidence. Due to wide variations in the measurement of variables affecting PMTCT service uptake, it was not feasible to conduct a meta-analysis to assess the effect of each factor. Hence, we performed narrative analysis after tabulating individual studies in terms of their characteristics. The quantitative data synthesis was guided by Andersen's behavioural model i.e. community factors including health facility factors, predisposing factors, enabling factors and need factors (55). Therefore, significant factors (as it was presented in the original paper) across studies were matched to the appropriate category in the Andersen behavioural model. In this review, meta-analyses were undertaken using R software version 4.2.1.

Result

Study identification

The studies included in this review were published from 01 January 2012 to 30 March 2022 and in English. A total of 2224 potentially relevant articles were retrieved from the literature search, with 1146 articles remaining after the deletion of duplicates. After title and abstract screening, 171 references were remained and included in the full-text screening. Finally, 52 studies were deemed eligible and included in this study (forty quantitative and eight qualitative and four mixed method articles) as illustrated with the PRISMA flow chart **[Figure 1].**

Figure 1. PRISMA flow chart displaying studies that were identified, screened and included in the review from databases.

Characteristics of included studies on prevention of mother-to-child transmission of HIV (PMTCT) services cascade

The studies published between 2012 and 2022 were carried out in a range of East African countries; namely Burundi (56), Comoros (56), Ethiopia (25, 45, 56-73), Kenya (56, 74-77), Malawi (23, 56, 78-80), Mozambique (81-83), Rwanda (56), South Sudan (84), Tanzania (85-89), Uganda (39, 90-92), Zambia (93, 94), and Zimbabwe (95, 96). Studies conducted among eligible pregnant/postpartum women, infants and service providers were included in the analysis. Almost all the studies (44/52) were facility-based (23, 25, 39, 45, 57-61, 63-74, 76-86, 88-99). Furthermore, the study design varied across studies: cross-sectional studies (n = 28); prospective cohort (n = 2); retrospective cohort (n=6), retrospective chart review (n=4), qualitative studies (n = 8) and mixed methods studies (n = 4). The mixed methods studies included a mix of surveys, interviews, and focus groups. The study characteristics are summarized in **[Table 1].**

Table 1: Characteristics of included studies on prevention of mother-to-child transmission of HIV (PMTCT) services (n = 52)

First Author, publication year and reference	Aim(s) of the study/ Phenomena of interest	Country and study period	Study Population and sample size	Study setting	Study design
Abtew 2015 (57)	To assess the acceptability of provider-imitated HIV testing and counselling (PITC) as an intervention for the PMTCT of HIV and to identify the associated factors	Ethiopia, 2014	• Pregnant women attending antenatal care (ANC) services (n=386)	Health facility	Cross-sectional
Ahoua 2020 (81)	To estimate the progress in the key indicators related to the PMTCT cascade in pregnant women enrolled in ANC services under Option B+	Mozambique, 2013-2017	• Pregnant women enrolled in ANC under Option B+ (n= 916280)	Health facility	Retrospective cohort
Akal 2018 (58)	To determine the status of PMTCT services utilization and factors affecting PMTCT utilization in health facilities of Afar region, Ethiopia	Ethiopia, 2014-2015	• Pregnant women attending ANC clinic (n=347)<br>• Health professionals who were providing PMTCT services (n=22)	Health facility	Cross-sectional
Alemu 2017 (25)	To identify the proportion of and factors for HIV testing among pregnant women in Ethiopia	Ethiopia, 2012-2013	• Pregnant mothers (n=416)	Health facility	Cross-sectional
Astawesegn 2021 (56)	To investigate the uptake and factors associated with prenatal HIV test uptake in East Africa.	Burundi 2016-2017, Comoros 2012, Ethiopia 2016, Kenya 2014, Malawi 2015-2016, Rwanda 2014-2015, Uganda 2016, Zambia 2013-2014, Zimbabwe 2015	• Mothers who had birthed two years before the surveys-Burundi (n= 5412), Comoros (n= 1298), Ethiopia (n= 4308), Kenya (n= 7357), Malawi (n= 6693), Rwanda (n= 3236), Uganda (n= 5901), Zambia (n= 5074), Zimbabwe (n= 2454)	Community	Cross-sectional
Augustine 2021 (95)	To evaluate the cascade of HIV care among the HEIs born in Mashonaland East Province of Zimbabwe	Zimbabwe, 2017	• HEIs (n=1028)	Health facility	Retrospective review
Bergmann 2017 (90)	To identify influences on access to and use of infant HIV health services, specifically nevirapine administration.	Uganda, 2014-2015	• HIV-positive pregnant women (n=384) and six FGDs (n=43)	Health facility	Mixed method
Berhan 2014 (59)	To assess the prevalence of HIV infection and associated factors among infants born to women living with HIV, in South Gondar zone, Amhara region, Ethiopia.	Ethiopia, 2013	• HEIs (n=434)	Health facility	Cross-sectional
Bobrow 2016 (78)	To understand barriers, facilitators and recommendations for five key steps in the EID and treatment cascade: (1) identification of	Malawi, 2013	• Mothers of HEIs (n=47)<br>• Healthcare workers (HCWs) providing EID and treatment (n=20)	Health facility	Qualitative

	HEIs; (2) infant testing; (3) sample processing and transport; (4) reporting results to mothers; (5) ART initiation for HEI.				
Buleza Lamucene 2022 (82)	To understand the perspectives of pregnant and postpartum women living with HIV in Sofala, Mozambique, regarding barriers and facilitators to following PMTCT recommendations	Mozambique, 2020-2021	• Pregnant and postpartum women living with HIV (n=15)	Health facility	Qualitative
Buregyeya 2017 (91)	To explore experiences of HIV-infected pregnant and breastfeeding women regarding barriers and facilitators to uptake and adherence to lifelong ART.	Uganda, 2014	• HIV-infected pregnant and lactating women. (n=57)	Health facility	Qualitative
Bwana 2018 (85)	To assess predictors of mothers/guardians to obtain EID services for children aged under 5 years exposed to HIV infection in Muheza district, Tanzania	Tanzania, 2015-2016	• Mothers/guardians with children below 5 years who were born to HIV-positive mothers and were not breastfeeding for ≥6 weeks (n=576).	Health facility	Cross-sectional
Cataldo 2017(79)	To explore the experience of patients and HCWs in relation to the implementation of Option B+.	Malawi, 2013.	• Nurses, medical assistants, and community health workers (Health Surveillance Assistants) (n=48)<br>• HIV-infected pregnant or breastfeeding women (n=24)	Health facility	Qualitative
Desta 2019(60)	To determine the prevalence and associated risk factors of HIV among HEIs in the Tigray regional state, Northern Ethiopia	Ethiopia, 2016.	• HEIs (n=340)	Health facility	Cross-sectional
Dzangare et al 2016(96)	To assess the uptake and success of Option B+ in pregnant and breastfeeding women in two rural districts of Zimbabwe.	Zimbabwe, 2014	• Women enrolled in ANC, labour and delivery care or post-natal care (n=2598)	Health facility	Retrospective cohort
Ebuy et al 2020(61)	To assess timely infant testing, testing for HIV at the 18th month, test results and factors influencing HIV positivity among infants born to HIV-positive mothers in public hospitals of Mekelle, Ethiopia.	Ethiopia, 2014-2017.	• Mother-infant pairs who were eligible for the PMTCT program (n=558)	Health facility	Cross-sectional
Ejigu et al 2018(62)	To assess the uptake of HIV testing during pregnancy and associated factors among Ethiopian women.	Ethiopia, 2016	• Women who were pregnant in the last year before the survey (n= 2414)	Community	Cross-sectional
Gaitho et al 2021(74)	To identify factors associated with timely uptake of virologic EID among HEI and gain insight into missed opportunities.	Kenya, 2015-2017	• HEIs (n=2020)	Health facility	Cross-sectional
Gamell et al 2017(86)	To describe the PMTCT cascade and uptake of Option B+ guidelines implemented through this service delivery model	Tanzania, 2014-2015	• Pregnant women (n= 1,579),<br>HEIs (n= 135)	Health facility	Prospective cohort
Gebeyehu et al 2019(63)	To assess the acceptance of HIV testing and associated factors among pregnant women	Ethiopia, 2019	• Pregnant women attending ANC (n=340)	Health facility	Cross-sectional

Gebremedhin et al 2018(64)	To describe the level of acceptance of PITC and associated factors among pregnant women attending 8 ANC clinics in Adama, Ethiopia.	Ethiopia, 2016	• Pregnant women attending ANC (n=441)	Health facility	Cross-sectional
Gebresillassie et al 2019(65)	To assess the utilization and acceptance rate of PICT as an intervention for PMTCT among pregnant women attending University of Gondar referral and teaching hospital, Ethiopia.	Ethiopia, 2018	• Pregnant women attending ANC (n=364)	Health facility	Cross-sectional
Haider et al 2022(75)	To examine the factors affecting HIV testing among women during pregnancy in Kenya.	Kenya, 2014	• Pregnant women (n=36,626)	Community	Cross-sectional
Hampanda et al 2017(93)	To explore how gender power dynamics within couples affect HIV-positive women's uptake of early infant HIV testing at a large health centre in Lusaka, Zambia.	Zambia, 2014	• HIV-positive mothers who had brought their child for routine pediatric immunizations (n=320)	Health facility	Cross-sectional
Kanguya et al 2022(94)	To understand readiness to start ART among HIV pregnant women from the perspectives of both women and men to suggest more holistic programs to support women to continue life-long ART after delivery.	Zambia, 2015	• HIV-infected pregnant women not yet on ART and HIV-infected pregnant or postnatal women on ART (n=20), Partners of women who were recently or currently pregnant (n=16)	Health facility	Qualitative
Kebede et al 2014(45)	To investigate the rate of EID and predictive factors of EID among infants born to HIV-infected women	Ethiopia, 2012	• Mother-infant pairs (n=266)	Health facility	Retrospective cohort
Konje et al 2018 (87)	To conduct a population-based study that examined the utilization and availability of ANC services	Tanzania, 2016-2017	• Pregnant women (n=1719)	Community	Mixed method
Lain et al 2020 (83)	To describe the completeness of follow-up until definitive diagnosis among HEI, who were enrolled in routine care, the presence of clinical events during follow-up and to analyse factors associated with LTFU and clinical events.	Mozambique, 2019	• HEIs (n= 1413)	Health facility	Retrospective cohort
Makau et al 2015 (76)	To evaluate determinants of EID and early treatment initiation among HIV-exposed children from informal settlements in Nairobi, Kenya.	Kenya, 2013	• HIV-infected mother-infant pairs (n= 238)	Health facility	Cross-sectional
Moges et al 2017 (66)	To determine the rate of HIV transmission and associated factors among HEIs in selected health facilities in East and West Gojjam Zones, Northwest Ethiopia	Ethiopia, 2015	• HEI-mother pairs (n=305)	Health facility	Retrospective cohort
Mukose et al 2021 (92)	To assess key issues around ART prescription and swallowing (uptake), early adherence and associated factors among HIV-	Uganda, 2013-2016.	• HIV-positive pregnant women (n=507), HIV-positive pregnant and breastfeeding women (n=57), Health provider(n=54)	Health facility	Mixed methods

	positive expectant women and lactating mothers on Option B+ in Central Uganda.				
Mustapha et al 2018 (39)	To evaluate the utilization of PMTCT services and associated factors among adolescent and young postpartum mothers aged 15 to 24 years at a public urban referral hospital in Uganda.	Uganda, 2015	• Postpartum mothers (HIV positive and negative) (n=418),<br>• For In-depth interviews (10 HIV positive and 10 HIV negative) postpartum mothers, For key informants (2 nurses, 2 counsellors, 3 peer educators, and 2 doctors)	Health facility	Mixed methods
Ng'ambi et al 2022 (80)	To describe HIV prevalence trends and assess the factors associated with the risk of HIV infection of HEI tested with DNA-PCR in Malawi between 2013 and 2020.	Malawi, 2013-2020	• HEIs (n=255 229)	Health facility	Retrospective review
Nungu et al 2019 (88)	To determine the re-testing uptake and the determinants of HIV re-testing in a rural region of Njombe in Tanzania.	Tanzania, 2015-2016	• Newly delivered mothers (≤7 days from delivery) (n=668)	Health facility	Cross-sectional
Ongaki et al 2019 (77)	To determine factors affecting uptake of PMTCT services among HIV-positive pregnant women at Lodwar County Referral Hospital in Turkana County, an arid area in northern Kenya.	Kenya, 2015-2016	• HIV-positive pregnant mother (n=230)	Health facility	Retrospective review
Oshosen et al 2021 (89)	To elicit the perspectives of PMTCT patients regarding the content and quality of the counselling they received during HTC in Tanzania.	Tanzania, 2016-2017.	• HIV-positive pregnant women (n=24)	Health facility	Qualitative
Tadewos et al 2020 (67)	To assess the proportion of mother-to-child transmission (MTCT) of HIV and associated factors among HEIs on follow-up in pastoralist health facilities, South Omo, Ethiopia.	Ethiopia, 2018	• HEI-mother pairs who were on follow-up care (n=228)	Health facility	Cross-sectional
Thidor et al 2019 (84)	To assess knowledge, attitude and practice of prevention of MTCT of HIV among pregnant women attending ANC at Juba Teaching Hospital, South Sudan	South Sudan, 2015	• Pregnant women (n=251)	Health facility	Cross-sectional
Tsehay et al 2019 (68)	To assess factors associated with HIV-positive serostatus among HEIs attending care at health facilities in Bahir Dar administration, Ethiopia	Ethiopia, 2018	• HEIs (n= 477)	Health facility	Cross-sectional
Van Lettow et al 2018 (23)	To estimate the use and outcomes of the Malawian programme for the MTCT of human immunodeficiency virus (HIV)	Malawi, 2014-2016	• Mother-infant pairs (n=33744)	Health facility	Cross-sectional

Wanyenze et al 2018 (97)	To assess the uptake of PMTCT services in a cohort of HIV-infected women in care at The AIDS Support Organization Jinja and Kampala in Uganda.	Uganda, 2013	• HIV-infected women with fertility intentions (n=299)	Health facility	Prospective cohort
Workagegn et al 2015 (69)	To identify predictors and possible barriers to HIV testing among ANC attendees based on the health belief model (HBM) in Addis Ababa, Ethiopia.	Ethiopia, 2013	• Pregnant women attending ANC (n=301)	Health facility	Cross-sectional
Wudineh et al 2016 (70)	To investigate mother-to-child<br><br>transmission (MTCT) of HIV infection and its determinants among HEIs on care at Dilchora Referral Hospital in Dire Dawa City Administration.	Ethiopia, 2013	• HEIs (n=382)	Health facility	Retrospective cohort
Zegeye 2020 (71)	To assess the PMTCT service utilization rate and to characterize its reasons among pregnant women attending ANC clinics at selected public health facilities in Debre Berhan Town, Northern Ethiopia.	Ethiopia, 2019	• Pregnant women attending ANC (n=355)	Health facility	Cross-sectional
Chadambuka et al 2018 (98)	To explore the acceptability of Option B+ among pregnant and lactating women in Zimbabwe	Zimbabwe, 2014-2015	• HIV-positive pregnant and breastfeeding women. (n=43 for In-depth interview, n=22 for Focus group interview)	Health facility	Qualitative
Semali et al 2014 (100)	To determine factors associated with uptake of HIV testing during ANC in Tanzania.	Tanzania, 2011-2012	• Women who attended antenatal clinic (ANC) and gave birth in the past two years (n=3555)	Community	Cross-sectional
Deressa et al 2014 (73)	To investigate factors associated with the acceptability and utilization of PMTCT of HIV.	Ethiopia, 2010	• Pregnant women attending ANC (n=843)	Health facility	Cross-sectional
Ford et al 2018 (101)	To investigate associations between decision-making and specific steps along the PMTCT cascade:	Zambia, 2011	• HIV-infected mother-infant pairs (n=344)	Community	Cross-sectional
Olana et al 2016 (72)	To assess the proportion of HIV-infected babies tested by DNA-PCR and factors affecting HIV transmission	Ethiopia, 2014	HEIs (n=624) and their mothers (n=412)	Health facility	Retrospective review
Yaya et al 2019 (102)	To assess the sociodemographic and economic factors associated with ANC use and HIV testing during pregnancy in Mozambique.	Mozambique, 2011	• Women who were pregnant in the last two years before the survey (N=7080)	Community	Cross-sectional
McCoy et al 2015 (103)	To examine the uptake of services and behaviours in the prevention of mother-to-child HIV transmission (PMTCT) cascade in Zimbabwe and determine factors associated with MTCT and maternal ART or ARV prophylaxis.	Zimbabwe, 2012	• Mothers/caregivers-infant's pair (n= 8,800)	Community	Cross-sectional
Kim et al 2016 (99)	To identify the main barriers and facilitators to uptake and adherence to ART under Option B+	Malawi, 2014	• HIV-positive pregnant and postpartum women (n= 65)	Health facility	qualitative

Uptake of PMTCT of HIV services in East Africa

We estimated pooled uptake for maternal HIV test, maternal ARV use, infant ARV prophylaxis and EID for the PMTCT of HIV in East Africa using a random effects model. The forest plots illustrated that the pooled uptake estimate was (82.69%; 95% CI: 75.62– 88.03 for maternal HIV test; 88.33%; 95% CI: 78.59–93.98 for maternal ART; 84.98%: 95% CI: 80.78-88.39 for infant ARV prophylaxis; 68.77%; 95% CI: 57.63-78.09 for EID) in East Africa **[Figure 2a-d].**

Figure 2a: Forest plot showing individual study and pooled estimates of Maternal HIV test service coverage in East Africa based on a random-effects model.

Figure 2b: Forest plot showing individual study and pooled estimates of Maternal ART coverage in East Africa based on a random-effects model.

Figure 2c: Forest plot showing individual study and pooled estimates of Infant ARV prophylaxis coverage in East Africa based on a random-effects model.

Figure 2d: Forest plot showing individual study and pooled estimates of early infant HIV diagnosis coverage in East Africa based on a random-effects model.

Subgroup analysis

In this review, subgroup analysis was conducted for the country, study setting and year of publication. Accordingly, Malawi (98.8%) and Uganda (95.7%) had the highest proportion of women tested for HIV, while South Sudan (72%) and Ethiopia (75.5%) had the lowest proportion of women tested for HIV. The highest proportion of women who initiated ART was found in Mozambique (97.66%) followed by Malawi (95.76%), whereas the lowest proportion of women who initiated ART was found in Zambia (68.8%) followed by Zimbabwe (78.7%). Of the eight countries that had data on infant prophylaxis and EID uptake, Zimbabwe had the highest infant prophylaxis (91.9%) and EID uptake (85.6%).

Moreover, consistent maternal HIV testing was found across years, however, the highest (93.78%) and the lowest (73.7%) proportion of maternal ART uptake was found between the year 2012-2014 and 2015–2017 respectively. In all PMTCT cascades, studies that were conducted in the community had lower rates (77.9%, 95% CI 69.7-84.3% for maternal HIV testing, 63.7%, 95% CI 53.6-72.8% for maternal ART uptake, 62.8%, 95% CI 60.1-65.3% for infant ARV prophylaxis) than studies that were conducted in the health facilities (84.7%, 95%

CI 72.9-91.9% for maternal HIV testing, 91%, 95% CI 82.6-95.6% for maternal ART uptake, 86.8%, 82.9-89.9% for infant ARV prophylaxis use **[S4 Table].**

Publication bias

To examine publication bias, we visually inspected the funnel plot shown in **[S1 Figure]**. A symmetric funnel plot indicates the absence of publication bias; that is, studies are evenly distributed on either side of the average effect size, regardless of the size of the study's sample. However, as shown in S1 Figure, the plots A to C are asymmetric. To test whether this asymmetry was statistically significant, we conducted Egger's regression test, which was significant for infant prophylaxis (z= 1.5725, p=0.001 for infant prophylaxis) but not for maternal HIV testing, maternal ARV uptake and EID (z = 0.4437, p = 0.6572 for maternal HIV testing, z = 1.3556, p = 0.1752 for maternal ARV uptake, z = -0.5671, p = 0.5706 for EID). Our further sensitivity analysis (i.e., trim-and-fill analysis) for infant prophylaxis did not reveal a significant influence on pooled uptake.

Reported factors associated with PMTCT of HIV services uptake in East Africa

Quantitative synthesis

Table 2 shows the factors that the quantitative studies have found to be statistically significantly associated with maternal HIV test, maternal ARV, infant ARV prophylaxis, and EID uptake. Factors from quantitative studies were organized into four categories according to Andersen's behavioural model such as community/health care, predisposing, enabling and need factors [**Table 2]**.

Community and health care factors: Six studies (25, 56, 57, 62, 65, 102) showed the presence of an association between place residence and maternal HIV testing. However, two studies on maternal HIV testing (64, 75) and three studies on EID (61, 85, 95) revealed the absence of statistical association with place of residence. Women who lived in a community where there was no/low stigmatized attitude toward people living with HIV/AIDS (25, 57) were more likely to be HIV tested than those who lived in a community that stigmatized HIV-positive patients (62, 75). Two studies explicitly indicated an association between the quality of available HIV testing services and maternal HIV test uptake (64, 88).

Predisposing factors: Eight studies reported the presence of an association (25, 56, 58, 62, 63, 65, 88, 102) and three studies (64, 69, 75) reported the absence of an association between educational level and maternal HIV testing. Of the eight studies that showed the presence of association, seven studies reported more educated women were found to be HIV tested than non-educated women (25, 56, 58, 62, 63, 88, 102). Similarly, for women who have educated

partners (56, 102) the odds of HIV testing were higher. Furthermore, a study conducted in Tanzania showed the presence of a correlation between children's age and marital status with EID (85).

Enabling factors: Factors under the enabling category included socioeconomic factors (for example, employment, higher monthly income and wealth index) (25, 56-58, 62, 65, 88, 102), access to the PMTCT services (56, 58, 85), knowledge/awareness about HIV/PMTCT/MTCT (25, 56, 62-64, 75, 85), exposure to media (56, 102), presence of support groups (90), and intimate partner violence (93). Studies showed that higher SES was positively associated with HIV testing. For example, having a higher wealth was positively related to taking HIV testing multiple times (56, 62, 102). Women who were employed (AOR = 2.1) (88), employed in the agricultural sector (AOR=1.325) (102), or government employees (AOR=2.15) (57) were more likely to have HIV testing in comparison to unemployed women. Three studies reported no significant association between occupation or employment and maternal HIV testing (56, 62, 63). Similarly, comprehensive knowledge about PMTCT is significantly associated with a higher rate of HIV testing (25, 56, 62, 63, 75). Only one Ugandan study reported associations between social support and infant ARV prophylaxis uptake (90) and mothers who are currently in a social support group were more likely to be HIV tested than their counterparts (AOR=2.50) (90). Nevertheless, women who have lower access to PMTCT service were less likely to use both maternal HIV testing and EID (56, 58, 85).

Need factors: Women who did not want to have more children were less likely to utilize maternal HIV testing (102). However, women who had favourable attitudes/benefits towards PITC (57, 63, 69) and more ANC visits (58, 64, 88) were found to be more likely to use the HIV test service. Women on ART at the time of the HIV PCR test and infants who had ARV prophylaxis at birth were positively associated with EID (45, 74). Likewise, factors such as ANC follow-up, birth at a government health facility (45), and Maternal ART adherence (93) were found to be positively correlated with EID. However, having an HIV-positive child resulted in lower odds of EID service uptake (85)

Table 2: Statistically significant factors affecting prevention of mother-to-child transmission of HIV cascade uptake from multivariable analyses in East Africa.

Cascades	Author, publication year and reference	Community/Health care factors*	Predisposing factors*	Enabling factors*	Need factors*	Controlled variables during multivariate analysis
Maternal HIV testing	Abtew 2015 (57)	• Residents: Rural (AOR=4.04), Urban (ref)<br>• Stigma toward people living with HIV/AIDS: No (AOR=3.54), Low (AOR=4.04), High (ref)		• Occupation: employed (AOR=2.15), students (AOR=6.00), merchants (AOR=4.43), housewives (ref)<br>• Planned to disclose their test results to their husbands: Yes (AOR=14.85), No (ref)	• Attitudes towards PITC: Favourable (AOR=1.57), Not favourable (ref)<br>• Perceived pre-test counselling service: Good (AOR=3.46), Poor (ref)	Partner reaction to the positive result, Access to health facilities for HIV testing, access to transport services, preferred sex, and age of counsellor.
	Alemu 2017 (25)	• Residents: Urban (AOR=3.42), Rural (ref)<br>• Favoured attitude toward persons living with HIV: Yes (AOR=2.42), No (ref)	• Women aged: 16–24 years (AOR=7.9), 25–29 years (AOR= 4.95), 30–34 years (AOR=3.31), ≥ 35 years (ref)<br>• Maternal education: secondary education and above (AOR=3.49), No formal education (ref)<br>• Parity: No child (AOR=4.34), 1–4 (AOR= 4.7), ≥5 (ref)	• Monthly expenditure: ≥30 USD (AOR=4.06), : < 30 USD<br>• Comprehensive knowledge about MTCT: Yes (AOR=3.73), No (ref)<br>• Comprehensive knowledge about PMTCT (AOR=2.56), No (ref)		No controlled variables
	Astawesegn 2021 (56)	• Resident: Rural (AOR=0.66), Rural (ref)<br>• Countries: Comoros (AOR= 0.007), Ethiopia (AOR = 0.04), Kenya(AOR=0.73), Malawi(AOR=0.73), Mozambique(AOR=0.15), Rwanda(AOR=2.30), Uganda(AOR=0.61), Zambia(AOR=0.54), Zimbabwe (AOR=0.42), Burundi (ref)	• Maternal education: primary education (AOR=1.29), secondary or higher education (AOR=1.96), No education (ref)<br>• Partners education: primary education (AOR=1.24), secondary or higher school (AOR=1.56), No education (ref)	• Distance to health facility: Challenging (AOR=0.8), Not challenging (ref)<br>• Wealth index: rich (AOR=1.57); middle AOR=1.29), Poor (ref)<br>• Read a magazine/newspaper: Yes (AOR=1.31), No (ref)<br>• Watch television: Yes (AOR=1.46), No (ref)<br>• Listen to the radio: Yes (AOR=1.13), No (ref)<br>• Awareness about MTCT of HIV during birth: Yes (AOR=1.73), No (ref)<br>• Awareness about MTCT of HIV during breastfeeding: Yes (AOR=1.41), No (ref)		Maternal age, Maternal occupation, History of Sexual violence, Household Decision making, Desire for pregnancy
	Ejigu 2018 (62)	• Stigmatizing attitudes towards HIV-positive people (AOR=0.57),<br>• Residence: Urban (AOR=3.30), Rural (ref)<br>• Region: Tigray (AOR= 9.55), Amhara (AOR=4.16), Addis Ababa/Harari/Dire Dawa (AOR= 3.55), Afar/ Somali (ref)	• Maternal education: Primary level (AOR=1.55), secondary level (AOR=2.56), higher level (AOR=3.95), No education (ref)<br>• Marital status: widowed/Divorced/Separated (AOR=0.07), Never married (ref)	• Women who were aware of MTCT during pregnancy (AOR=2.03),<br>• Wealth index: Poorer (AOR=2.32), Middle (AOR=3.27), Richer (AOR=5.43), Richest (AOR=5.84), Poorest (ref)<br>• Aware of MTCT during pregnancy: Yes (AOR= 2.03), No (ref)		Maternal age, Religion, Employment status

		• Stigma attitude towards HIV-positive people: Yes (AOR=0.57), No (ref)				
	Gebeyehu 2019 (63)		• Educational status: No formal education (AOR= 0.393), primary school (AOR=0.357), Tertiary school and above (ref)	• Awareness about MTCT: Yes (AOR=2.602), No (ref) • Knowledge of PMCT: Knowledgeable (AOR=1.715), not Knowledgeable (ref)	• Perceived the benefit of HIV testing: Yes (AOR=1.838), No (ref) • Number of ANC visits: Two or more ANC visits (AOR=2.485), One visit (ref)	Occupation, heard from a friend, heard from a health provider
	Akal 2018 (58)		• Educational status: could read and write (AOR=11.3), could not read and write (ref)	• Income level per month: ≥1000 ETB (AOR=5.6), <1000 ETB (ref) • Access to the PMPCT service: Yes (AOR=6.5), No (ref)		Knowledge about PMTCT service
	Gebremedhin 2018 (64)	• Quality of PITC service (AOR=1.91)	• Older age (AOR=0.37)	• Higher level of knowledge on MTCT (AOR =1.82), • Negative partner attitude toward positive HIV result (OR=0.31),	• More ANC visits (AOR=2.59)	Residence, educational status, monthly income, availability of HI V testing and counselling service, gender preference of the counsellor, stigmatizing attitude
	Gebresillassie 2019 (65)	• Resident: Rural (AOR=3.64), urban (ref)	• Educational status: No formal education (AOR=3.15), Primary education status (AOR=2.73), tertiary education (ref)	• Higher average monthly income (AOR=4.01), • Employment status: self-employed (AOR=0.31), Unemployed (ref) • Planned to disclosure HIV test results to male partners: Yes (AOR=7.81), No (ref)		Client age in year, Religion, Parity, Partner reaction to a positive result, Attitude toward PICT
	Haider 2022 (75)	• HIV-related stigma (AOR=0.83), • Regions: North Eastern (AOR=0.33), Eastern (AOR=0.53), Central (AOR=0.45), Rift Valley (AOR=0.58), Western (AOR=0.42), Coast (ref) • HIV counselling during ANC visits: Yes (AOR=1.89), No (ref)	• Maternal age: 20–29 years (AOR=0.51), 30–39 years (AOR=0.31), 40–49 years (AOR=0.35), 15–19 years (ref) • Marital Status: Married and living with a partner (AOR=1.48), Never in union (ref)	• Knowledge about HIV Transmission during pregnancy (AOR=1.19)		Maternal education, Religion, Residence, and Wealth Index.
	Nungu 2019 (88)	• Quality of HIV testing: Yes (AOR=2.1), No (ref)	• Education: High Education (AOR=1.9), Low Education (ref)	• Employment status: employed (AOR=2.1), unemployed (ref)	• Reported symptoms of sexually transmitted infections: Yes (AOR=4.9), No (ref) • Number of ANC visit: ≥4 visits (AOR=1.8), < 4 (ref) • Ever used condom: Yes (AOR=1.7), No (ref)	Parity, Perceived severity of HIV
	Workagegn 2015 (69)		• Maternal age: 21-25 years (AOR=2.43), 26-30 years (AOR=2.3), > 35 years (ref)		• Perceived net benefits: low (AOR=0.34), high (ref) • Perceived self-efficacy: high (AOR=1.90), low(ref)	Educational status, Marital status, Perceived threat, Cues to action
	Yaya 2019 (102)	• Resident: Rural (AOR=0.788), urban (ref) • Ethnicity: Portugais AOR=2.098), Xichangana (AOR=2.072), Cisena (AOR=1.381),Elomwe(AOR=0.418), Cindau (AOR=1.415),	• Maternal age (AOR=1.232 for age 20–24, AOR=1.422 for age 25–29, AOR=1.376 for age 30–34, for age 35-39, AOR= 1.283, age 15–19 (ref)),	• Employment: agricultural (AOR=1.325), Professional/Technical/Managerial employed (AOR=0.861), Not Working (Not working (ref)	• Wanted more child: No (AOR=0.643), Yes (ref)	Parity, Household head’s sex

		Xitswa(AOR=6.637), Other(AOR=3.168), Emakhuwa (ref)<br>• Religion: Other (AOR=0.868), Islam (ref)	• Maternal education: primary (AOR=1.373), secondary (AOR=1.394), no education(ref)<br>• Husband education: primary (AOR= 1.225), secondary (AOR=1.453), no education(ref)	• Wealth quantile: Middle (AOR=1.241), richer (AOR=1.429), richest (AOR=1.959), Poorest (ref)<br>• Media access: Yes (AOR=1.233), No (ref)		
Infant ARV prophylaxis	Bergmann 2017 (90)			• Involving in the support group (AOR=2.50)	• Delivered at home (AOR=0.02)<br>• Mothers who took < 95 % of ART doses in the last 30 days (AOR=3.55),	Maternal age, marital status, education, number of children, disclosure of HIV status to partner, and health care facility,
Early infant diagnosis	Augustine 2021 (95)				• Mode of delivery: Non-normal vertex delivery (RR=1.19), normal vertex delivery (ref)	Sex, Place of birth, Birth weight (g), Place of residence, Type of health facility, District.
	Bwana 2018 (85)		• Married/living together with their spouses (AOR=2.3), Separated/divorced/widow (ref)<br>• Child age in months: 13–59 months (AOR=0.4), ≤12 months (ref)	• Living far away from the health facility (AOR=0.6),<br>• Mothers with good knowledge of HIV (AOR=2.4)	• A child being found HIV positive (AOR=0.3)	Sex, residence, Place of delivery, Maternal HIV status at conception, planned pregnancy, age of the household head, household head Educational level, Monthly income, size of the household
	Ebuy 2020 (61)	• Counselling on feeding options: yes (AOR=2.01), no (ref)			• ANC follow-up: Yes (AOR=2.77), no (ref)	Place of residence, Mother WHO clinical stage, Current CD4 count of mothers, Mothers' adherence status, Provision of postpartum family planning, Infants birth weight, Infant ARV prophylaxis (NVP syrup) given
	Gaitho 2021 (74)				For late HIV diagnosis<br>• Mothers on ART at the time of HIV PCR test: No (AOR=1.27), yes (ref)<br>• Received infants ARV prophylaxis: no (AOR=1.43), yes (ref)	Sex, Birth weight, Mode of delivery, Place of delivery
	Hampanda 2017 (93)			• Intimate partner violence (AOR=0.41),<br>• Disclosed HIV status to male partner (AOR=13.73)	• Maternal ART adherence (AOR=2.28),	Maternal age, Parity, completed secondary education, Standardized wealth Index, Diagnosed with HIV during most recent pregnancy, Male partner HIV status:
	Kebede 2014(45)				• Mothers on HAART during pregnancy (AOR=3.4)<br>• Received ARV prophylaxis during pregnancy (AOR=3.7)<br>• Place of birth: Government health facility (AOR= 2.9), Home (ref)	Prenatal care (ANC), the Time mother gets HIV diagnosed, Mother prophylaxis at labour, Infant prophylaxis at birth

*AOR- Adjusted Odds Ratio, RR-Relative Risk, * The factors included only significant factors adjusted for confounders*

Qualitative synthesis

Qualitative studies and qualitative aspects of mixed methods studies were thematically analysed. Themes such as health care factors, access to services, partner-related factors, acceptance and disclosure of HIV status, stigma and misconception, knowledge and couples' differences in HIV status, disease progression, fear related to ART and good health were generated **[Table 3].**

Health care factors: Most women and providers believed that health system factors were the most common challenges across PMTCT cascades. This review illustrated how lack of privacy (78, 79, 87, 89), shortage of staff and HIV test kits (79), negative attitude/behaviour of health workers (39, 90), lack of age-specific service (39), long waiting times (39) and lack of supervision (78) were often responsible for low PMTCT services uptake.

Lack of privacy and counselling were crucial factors, particularly during maternal HIV testing and ART initiation (79, 87, 89), Furthermore, the shortage of healthcare workers (HCWs) in comparison to the number of clients and the irregular availability of HIV test kits were both reported as a challenge (39). Providers also commonly reported that lack of follow-up supervision for nurses working in the ART clinics, antenatal clinics (ANCs) and maternity wards, as well as for laboratory and health surveillance assistants who provide EID and treatment services as a challenge (78). Likewise, the absence of a reliable transport system for DBS to perform DNA PCR tests for EID at central reference laboratories was also identified as a challenge. It requires specimen transport over long distances from health centres to the central labs where samples are analysed. The long waiting time between DBS sample collection and the processing of results meant there were repeated facility visits by women and associated unnecessary transport costs. This created frustration and worry in women about the health of their children (78).

A range of healthcare-related motivators was described such as free treatment/services, peer support, client motivation emotional support in health facilities, implementation of SMS and a good referral system.

Access to facilities/services: Long distances to the health facility (39, 79) and financial constraints (39, 82) were reported by women as a barrier to accessing maternal HIV testing and ART initiation. However, a study done in Uganda reported that having access to free treatment and services as a strong motivator for the uptake of PMTCT services (39).

Partners-related factors: Domestic violence by a partner, lack of partner support, and blame when using ART (39, 79, 82, 90, 91, 94, 99) were reported by women. Women feared domestic their partner violence and were not happy to disclose their HIV status and initiate treatment

(79, 91). Likewise, women were usually frustrated with the continuity of their relationship if their partners know they are HIV positive and on ART (79, 91, 94, 99).

Stigma and misconceptions: This review illustrated that stigma and misconceptions remain central concerns for women accessing PMTCT services. Five qualitative studies with a focus on HIV testing and ART uptake among pregnant women suggested that HIV-related stigma and misconceptions prevented them from service use (39, 79, 82, 91, 92). Women would be stigmatized when they are seen testing for HIV and taking ARV medication. In addition, the embodied misconception such as traditional health beliefs and practices (82, 92) as well as religious views (79, 82, 91) towards HIV played a negative role in the uptake of HIV testing and ART.

Knowledge and couples' difference in HIV status: women lacking comprehensive knowledge about HIV and the benefits of ART were identified as a barrier (39, 89, 92). Moreover, when couples in which the women were infected but the men were HIV-negative, the woman usually experiences emotional and psychological distress (94). This emotional and psychological distress delayed their acceptance and initiation of ART. Therefore, when women are in this situation, counselling support is highly needed to encourage them to disclose their HIV-positive status to their HIV-uninfected partners and utilize the services.

Disease progression: women who acquire HIV does not show the sign and symptoms of the disease and women are usually asymptomatic/clinically healthy at the early stage of HIV infection usually at the time of diagnosis (82, 99). Hence, when women do not have the sign of AIDS diseases, they feel that they are healthy and would not accept their HIV-positive status and initiate ART treatment as early as possible to prevent vertical transmission.

Fear related to ART: Our review also observed that women do not initiate ART because of the fear they have towards ART drugs; such as fear of potential ART side effects (3 studies) (39, 82, 92), fear of lifelong commitment to taking ART (4 studies) (39, 91, 92, 99), and fear of its size (1 studies) (91). The studies done in Uganda, (39, 91, 92) and Malawi(99) reported that most mothers had fears of taking ART treatment daily for lifelong medications which deter them from starting the treatment. Furthermore, women perceived that if they are not adherent after once the treatment have started they would die quicker, so they did not want to start treatment at all since they were not sure that they would be adherent (91).

Good health: Women's desire to be healthy (91, 92, 98) and an interest to protect their unborn children from acquiring HIV infection (39, 91, 92, 94, 98) weres identified as strong motivators to initiate ART. These studies indicated that the interest of women to be healthy and to have

an HIV-free child inspired them to initiate ART uptake because mothers who are highly concerned about their unborn child are more likely to utilize the services hoping that the child would be found negative in the end.

Table 3: A summary qualitative synthesis of factors to PMTCT services uptake in East Africa

	**Author, publication year, and reference number**												
	Konje 2018(87)	Oshosen 2021(89)	Cataldo 2017(79)	Mustapha 2018(39)	Buleza Lamucene 2022(82)	Mukose 2021(92)	Buregyeya 2017(91)	Kanguya 2022(94)	Kim et al 2016(99)	Chadambuka 2018(98)	Bergmann 2017(90)	Bobrow 2016(78)	**Total**
**HIV testing**													
**Healthcare-related factors**													
Lack of privacy and inadequate counselling	✓	✓											2
Shortage of Staff and HIV test kits			✓										1
The negative attitude of health workers				✓									1
Long waiting time at the clinic				✓									1
Lack of age-specific service for adolescent				✓									1
Emotional Support by nurses*		✓											1
**Access to facilities/services**													
Long distance to the health facility				✓									1
**Acceptance of HIV status**				✓									
Fear or denial to accept that they are HIV-positive	✓	✓		✓									3
**Stigma and misconceptions**													
Belief witchcraft is the origin of the disease					✓								1
Stigma and discrimination				✓									1
**Disease progression**													
Being asymptomatic state				✓	✓								2
**Maternal ART uptake**													
**Healthcare-related factors**													
Lack of privacy/confidentiality in ART services			✓										1
Motivation by a health worker to start treatment*							✓						1
Time to ART initiation (Same day ART initiation) *			✓						✓				2
Support and counselling by peer educators *				✓	✓								2
Support and counselling/health education by health professionals*				✓		✓	✓			✓			4
**Access to facilities/services**													
Long distance to a health facility			✓										1
Financial constraints				✓	✓								1
Access to free treatment and services*				✓									1
**Partners related factors**													
Domestic violence and abandonment			✓				✓						2
Lack of partners' support				✓	✓			✓					3
Partner blame or divorce.			✓				✓	✓	✓				4
**Acceptance and disclosure of HIV status**													
Doubt of HIV positive results.						✓							1
HIV status discloser to their partners										✓			1
**Stigma and misconceptions**													
Religious belief			✓		✓		✓						3
Preference for local herbs.						✓							1
HIV-related stigma and discrimination			✓		✓		✓						3
**Knowledge and couples' differences in HIV status**													

Lack of knowledge about the purpose of ART		✓		✓		✓								3
Knowing the benefits of ART*						✓	✓			✓				3
Discordant couples								✓						1
**Disease progression**														
Asymptomatic state/feeling healthy					✓				✓					2
**Fear related to ART**														
Fear to take ART for lifelong				✓		✓	✓		✓					4
Fear of drug side effects				✓	✓	✓								3
Fear of the big size of the tablets							✓							1
**Good Health**														
Mothers desire to have an HIV-negative baby*				✓		✓	✓	✓		✓				5
Mother desires to remain healthy*						✓	✓			✓				3
**Infant ARV prophylaxis**														
**Healthcare-related factors**														
The bad behaviours of healthcare workers											✓			1
Financial and educational benefits that support groups provided*											✓			1
**Partners related factors**														
Lack of partner support											✓			1
**Early infant diagnosis**														
**Healthcare-related factors**														
Lack of follow-up supervision												✓		1
Lack of space at health centers												✓		1
Good referrals and coordinating system*												✓		1
Community health education about EIDT*												✓		1
New PMTCT guidelines (enrolling HEIs into the HIV care system immediately after delivery) *												✓		1
SMS system to receive HIV results*												✓		1
Follow-up system at the health center*												✓		1
No reliable transport system, long distance to facility, lack of fuel for specimen transport, and cost of transport												✓		1
**Acceptance and disclosure of HIV status**												✓		1
Refusal of HIV status												✓		1
Lack of HIV status disclosure to their partners												✓		1
Women/child health records												✓		1
Do not bring relevant health information, including HIV status, to the health facilities												✓		1

*facilitating/motivating factors

Discussion

This systematic review aimed to investigate the uptake and determinants of PMTCT of HIV services in East Africa. Accordingly, the overall pooled proportion was (82.69% for maternal HIV testing; 88.33% for maternal ART uptake; 84.98% for infant ARV prophylaxis; and 68.77% for EID) in East Africa. The finding was found to be promising, however, much work remains to be done to achieve the UNAID's target by 2030 (8). Besides, in comparison with the other PMTCT cascades, lower EID uptake was observed. This could be due to its complexity as it requires molecular techniques to detect viral nucleic acid rather than serological methods (42). Trained staff and complex laboratory equipment are required to perform dried blood spot (DBS) samples at central reference laboratories. This is challenging due to resource-limited settings and specimen transport over long distances and prolonged turn-around time for the return of results after testing has been performed (42, 104).

The findings of the sub-group analysis showed variation in the level of uptake from country to country. This could be due to differences in socioeconomic characteristics, early adoption of WHO recommendations into the national health system (48), and availability and quality of the service between countries (105-107). Besides, in comparison with community-based studies higher uptake was observed in facility-based studies as facility settings studies are expected to involve women who have access to health services with ongoing PMCT services and awareness programs.

The quantitative component of the review pointed to a wide range of community /health care factors (such as place of residence/geographical location, stigma, quality of PMTCT services), and predisposing factors (such as maternal age, child age, education status, religion, marital status, household size, ethnicity, partner attitude, occupation status). Along with enabling factors (such as income, distance to a health facility, decision-making power) and need factors (ANC follow-up, ART adherence, child HIV status, desire to have a child, perceived benefits/ self-efficacy of PMTCT, STIs symptoms, Parity). Several factors determining the PMTCT services that were identified by the quantitative studies were reflected in the synthesized findings from the included qualitative studies. These included lack of access to facilities/services, disclosure of HIV status, lack of age-specific service, stigma, and lack of knowledge about PMTCT services. The qualitative findings also pointed to additional factors not identified by the quantitative studies. These included the shortage of resources, lack of follow-up supervision, lack of privacy and confidentiality, fear related to ART, being asymptomatic and being a discordant couple.

Amongst the community factors, residence was associated with maternal HIV testing (25, 56, 57, 62, 65, 102). However, its effect was inconsistent in that Alemu et al. (25), Astawesegn et al. (56), Yaya et al. (102) and Ejigu et al. (62) found urban residents were more likely to be HIV tested, whilst Abtew et al. (57) and Gebresillassie et al. (65) revealed rural residents were more likely to be HIV tested. The finding of increased maternal HIV testing among urban mothers may be due to the availability and access of more healthcare centers, and a shorter distance to these centers in urban areas in comparison with rural areas (108, 109). Whereas, the second finding could be women from these rural areas may not opt-out from HIV testing, because women from rural areas believe that their doctor/nurse will react negatively to their refusal unless adequately informed about the opt-out policy by healthcare professionals (110). This review also identified stigma and discrimination as the most prominent barrier deterring women from seeking HIV test uptake. Social stigmatization may result in difficulty to attend regular clinic visits and further reduces their opportunities for a social support system that facilitates disclosure of their HIV status and the subsequent decision to take PMTCT services. Therefore, efforts should be directed at community education and sensitization about HIV to change communities' views about HIV from a fearsome death sentence to a manageable chronic condition (111).

Likewise, among the predisposing factors, never-married, widowed, or divorced women were less likely to take both maternal and infant HIV test services in comparison to their married counterparts (62, 75, 85). This may be linked to the lack of psychosocial and financial support that husbands may provide (112). Moreover, being widowed/divorced/separated women is socially unacceptable in most developing communities (112, 113) and therefore women may fear discrimination and feel ashamed of receiving PMTCT services (112).

There is an interplay between higher educational attainment for women and their husbands/partners and higher SES (employment, income, and wealth index status) in influencing maternal HIV test uptake (25, 56, 58, 62, 65, 88, 102). This is because, the more educated women are, the greater their level of employment and financial independence and the better informed they are about the importance of PMTCT services (114). A similar effect of SES on maternal and child health service use has been documented in other studies (115, 116). The more a community is advantaged socioeconomically, the higher the likelihood of women in that community utilizing health services (117, 118). This finding highlights the need to promote universal primary education and improve women's SES.

The present review demonstrated that knowledge and awareness about HIV and PMTCT were significantly associated with maternal HIV testing (25, 56, 62-64, 75) and EID (85). Individuals' knowledge and awareness of health issues influenced their: perceived need, perceived services benefit and recognition of the healthcare service centres which provided health services. Therefore, repeated PMTCT messages through media to improve women's knowledge and motivate them to use PMTCT services for themselves and their children, especially in developing countries is recommended (119). Whereas, long travelling distances to access health facilities providing PMTCT services were associated with lower odds of PMTCT services utilization (56, 58, 85), because, during pregnancy, walking or travelling long distances is very difficult and may discourage women from using the services in addition to travel-related costs.

Regarding the association between need factors and PMTCT service uptake, there was clear evidence for a positive association between these factors. In line with studies conducted in Ghana (120), and Cameroon (121), ANC follow-up and facility delivery were found to be important factors associated with PMTCT service uptake (61, 63, 64, 88, 90). ANC follow-up and facility delivery create an opportunity for early screening and enrolment of mothers and their newborns into the PMTCT service (19). Furthermore, we found that a child who has used ARV prophylaxis (45, 74) and was born from a mother who took ART/HAART (74) had higher odds of testing for HIV at six weeks. Women on ART and infants on ARV prophylaxis are more likely to access EID services because of good awareness of their HIV status, more frequent medical visits, and increased opportunities for providers to identify HEIs. Therefore, attendance and integration of maternal and child health (MCH) services (e.g. ANC) with the PMTCT program is an important strategy to eliminate HIV infection among children (75).

Concerning findings from the qualitative component, we found healthcare factors (such as shortage of resources, lack of privacy and confidentiality, and lack of follow-up supervision) as a barrier. For example, staff shortages may mean that healthcare providers are unable to provide services to all women in need, which can be experienced directly by women as neglected. Poor infrastructure may also create stressful working environments, which may predispose healthcare providers to behave poorly towards women (79, 87). Similarly, the absence of follow-up supervision was also identified by HCWs as a challenge in EID service delivery. If HCWs are not supervised and trained, they may not have the necessary skills or knowledge of current treatment protocols or referral procedures for providing EID. Hence, ensuring closer supervision of health workers and provision of the needed work inputs and training could improve the utilization of PMTCT services. Furthermore, consistent with a

previous systematic review (122), fear related to ART, being asymptomatic and being a discordant couple have been also associated with lower ART initiation. Whereas same-day ART initiation, support/counselling/motivation by health workers or peer educators, free treatment/services, mother's wish to be healthy or to have an HIV-free child, application of SMS system, good referrals and coordinating systems were identified as facilitators or motivators. It is important to note that even though same-day ART initiation is an important factor to increase ART initiation it has been reported to result in poor adherence and treatment discontinuation (122), because women may not have adequate time to think and make an informed decision before initiating ART.

This systematic review has several strengths, firstly, we used a comprehensive approach to capture all possible articles on this review question. Secondly, we have included articles conducted with quantitative, qualitative, and mixed-methods study designs, without restricting any study design/method. This approach was taken as it is widely regarded as best practice to provide decision-makers with information to inform policy-making (54). Thirdly, we explored whether there were differences in the level of uptake and factors across key PMTCT cascades. As a limitation, firstly, we used a narrative synthesis of factors associated with PMTCT cascades due to high heterogeneity among the studies. Secondly, we did not search for grey literature, we did not consider publications in other languages apart from English. Thirdly, we only examined the uptake and factors related to maternal HIV test, maternal ART use, infant prophylaxis, and EID of PMTCT cascade.

Conclusion

In conclusion, the pooled uptake of the PMTCT service cascade was promising in East Africa, but this finding should be interpreted with caution mainly because of the high between-study variability. Ensuring women and their children are enrolled and retained across the PMTCT cascade is recommended. The most identified factors associated with the service uptake were residence, maternal education, SES, stigma towards HIV-positive women, lack of knowledge on PMTCT, intimate partner violence, and lack of access to PMTCT service. These factors are modifiable if we give attention to address systematically at both policy and service provision levels. Therefore, promoting education, and economic empowerment, strengthening staff supervision, improving access and integration with maternal and child health services, and reducing stigma with active community participation are recommended to ensure HIV-free generations.

List of abbreviations

AIDS: Acquired Immune Deficiency Syndrome; ANC: Antenatal care; AOR: Adjusted Odds Ratio; ART: Antiretroviral therapy; ARV: Antiretrovirals; CI: Confidence Interval; EID: Early Infant HIV Diagnosis; HCWs: Health Care Workers; HEIs: HIV Exposed Infants; HIV: Human Immunodeficiency Virus; MCH: Maternal and child health; MMAT: Mixed Methods Appraisal Tool; MTCT: Mother-To-Child Transmission of HIV; PITC: Provider Initiated HIV Testing and Counselling; PMTCT: Prevention of mother-to-child transmission of HIV; SES: socioeconomic status; SSA: Sub-Saharan Africa; UNAIDS: The United Nations Programme on HIV and AIDS

Declarations

Ethics approval and consent to participate

Not applicable.

Consent for publication

Not applicable.

Availability of data and materials

All relevant data are within the manuscript and its Supporting Information files.

Competing interests

The authors declare that they have no competing interests.

Funding

No funding was obtained for this study.

Author contributions

Conceptualization: Feleke Hailemichael Astawesegn, Elizabeth Conroy, Haider Mannan, Virginia Stulz.

Data curation: Feleke Hailemichael Astawesegn.

Formal analysis: Feleke Hailemichael Astawesegn, Virginia Stulz.

Methodology: Feleke Hailemichael Astawesegn, Elizabeth Conroy, Haider Mannan, Virginia Stulz.

Project administration: Feleke Hailemichael Astawesegn, Elizabeth Conroy

Supervision: Elizabeth Conroy, Haider Mannan, Virginia Stulz

Writing – original draft: Feleke Hailemichael Astawesegn.

Writing – review & editing: Feleke Hailemichael Astawesegn, Elizabeth Conroy, Haider Mannan, Virginia Stulz.

Acknowledgment

The authors would like to thank Mr Tesfaye Yitna from Wolaita Sodo University, School of Nursing for his assistance in the study selection.

References

1. Edmonds A, Yotebieng M, Lusiama J, Matumona Y, Kitetele F, Napravnik S, et al. The Effect of Highly Active Antiretroviral Therapy on the Survival of HIV-Infected Children in a Resource-Deprived Setting: A Cohort Study. Plos Med. 2011;8(6).
2. Global Health Policy. The Global HIV/AIDS Epidemic 2022 [cited 2023 25 February]. Available from: https://www.kff.org/global-health-policy/fact-sheet/the-global-hivaids-epidemic/
3. Chi BH, Bolton-Moore C, Holmes CB. Prevention of mother-to-child HIV transmission within the continuum of maternal, newborn, and child health services. Current Opinion in HIV and AIDS. 2013;8(5):498.
4. Kiragu K, Collins L, Von Zinkernagel D, Mushavi A. Integrating PMTCT into maternal, newborn, and child health and related services: experiences from the global plan priority countries. JAIDS Journal of Acquired Immune Deficiency Syndromes. 2017;75:S36-S42.
5. (UNAIDS) JUNPoHA. GLOBAL PLAN TOWARDS THE ELIMINATION OF NEW HIV INFECTIONS AMONG CHILDREN BY 2015 AND KEEPING THEIR MOTHERS ALIVE. 2011.
6. Joint United Nations Programme on HIV/AIDS. UNAIDS DATA . UNAIDS; 2019 [cited 2022 May 2]. Available from: https://www.unaids.org/sites/default/files/media_asset/2019-UNAIDS-data_en.pdf.
7. Neary J, Langat A, Singa B, Kinuthia J, Itindi J, Nyaboe E, et al. Higher prevalence of stunting and poor growth outcomes in HIV-exposed uninfected than HIV-unexposed infants in Kenya. Aids. 2022;36(4):605-10.
8. The United Nations Programme. On the fast-track to an AIDS-free generation. The incredible journey of the global plan towards the elimination of new HIV infections among children by 2015 and keeping their mothers alive 2016 [cited 2021 26 May]. Available from: https://www.unaids.org/sites/default/files/media_asset/GlobalPlan2016_en.pdf
9. World Health Organization. Global guidance on criteria and processes for validation: Elimination of mother-to-child transmission of HIV and Syphilis, Second edition Geneva, Switzerland WHO; 2017 [cited 2021 2 January]. Available from: https://apps.who.int/iris/bitstream/handle/10665/259517/9789241513272-eng.pdf?sequence=1
10. UNICEF, UNAIDS, WHO . Key considerations for programming and prioritization. going the 'LAST MILE' to EMTCT:: A road map for ending the HIV epidemic in children 2020 [cited 2021 2 January]. Available from: http://www.childrenandaids.org/sites/default/files/2020-02/Last-Mile-To-EMTCT_WhitePaper_UNICEF2020.pdf
11. World Health Organization. Programmatic update: use of antiretroviral drugs for treating pregnant women and preventing HIV infection in infants: executive summary. 2012 [cited 2022 14 April]. Available from: https://apps.who.int/iris/bitstream/handle/10665/70892/WHO_HIV_2012.6_eng.pdf?sequence=2&isAllowed=y
12. World Health Organization. Prevention of mother-to-child transmission (PMTCT) 2018 [cited 2020 25 January]. Available from: https://www.who.int/gho/hiv/epidemic_response/PMTCT_text/en/
13. Center for Disease Control and Prevention. Prevent mother to child transmission CDC; 2020 [cited 2021 2 May]. Available from: https://www.cdc.gov/hiv/basics/hiv-prevention/mother-to-child.html
14. D'Ippolito M, Read JS, Korelitz J, Joao EC, Mussi-Pinhata M, Rocha N, et al. Missed opportunities for prevention of mother-to-child transmission of human immunodeficiency virus type 1 in Latin America and the Caribbean: the NISDI perinatal study. Pediatr Infect Dis J. 2007;26(7):649-53.
15. Joint United Nations Programme on HIV/AIDS. Global HIV/AIDS response epidemic update and health sector progress towards universal access 2011 [cited 2020 25 January]. Available from: https://www.unaids.org/sites/default/files/media_asset/20111130_UA_Report_en_1.pdf.

16. Joint United Nations Programme on HIV/AIDS: UNAIDS and PEPFAR announce dramatic reductions in new HIV infections among children in the 21 countries most affected by HIV in Africa 2016 [Available from: https://www.unaids.org/sites/default/files/20160608_PR_GlobalPlan_en.pdf [accessed on 21 January 2021].
17. Hamilton E, Bossiky B, Ditekemena J, Esiru G, Fwamba F, Goga AE, et al. Using the PMTCT cascade to accelerate achievement of the global plan goals. Journal of acquired immune deficiency syndromes (1999). 2017;75(1):S27.
18. Suthar AB, Hoos D, Beqiri A, Lorenz-Dehne K, McClure C, Duncombe C. Integrating antiretroviral therapy into antenatal care and maternal and child health settings: a systematic review and meta-analysis. B World Health Organ. 2013;91(1):46-56.
19. Turan JM, Steinfeld RL, Onono M, Bukusi EA, Woods M, Shade SB, et al. The Study of HIV and Antenatal Care Integration in Pregnancy in Kenya: Design, Methods, and Baseline Results of a Cluster-Randomized Controlled Trial. PLoS One. 2012;7(9) (no pagination).
20. Oladokun R, Awolude O, Brown B, Adesina O, Oladokun A, Roberts A, et al. Service uptake and performance of the prevention of mother-to-child transmission (PMTCT) programme in Ibadan, Nigeria. 2010.
21. Dionne-Odom J, Welty TK, Westfall AO, Chi BH, Ekouevi DK, Kasaro M, et al. Factors associated with PMTCT cascade completion in four African countries. AIDS research and treatment. 2016;2016.
22. Wettstein C, Mugglin C, Egger M, Blaser N, Vizcaya LS, Estill J, et al. Missed opportunities to prevent mother-to-child-transmission: Systematic review and meta-analysis. Aids. 2012;26(18):2361-73.
23. Van Lettow M, Landes M, Van Oosterhout J, Schouten E, Phiri H, Nkhoma E, et al. Prevention of mother-to-child transmission of HIV: a cross-sectional study in Malawi. B World Health Organ. 2018;96(4):256.
24. Ajayi A, Awopegba O, Owolabi E, Ajala A. Coverage of HIV testing among pregnant women in Nigeria: progress, challenges and opportunities. Journal of public health (Oxford, England). 2021;43(1):e77-e84.
25. Alemu YM, Ambaw F, Wilder-Smith A. Utilization of HIV testing services among pregnant mothers in low income primary care settings in northern Ethiopia: a cross sectional study. BMC Pregnancy & Childbirth. 2017;17:1-8.
26. Morales JA, Hamahuwa M, Moyo N, Mutanga JN, Schue JL, Maunga S, et al. Factors associated with antiretroviral therapy use among pregnant women in rural and urban settings in Southern Province, Zambia: 2016–2019. Tropical Medicine & International Health. 2022;27(10):902-12.
27. Valle S, Pezzotti P, Floridia M, Pellegrini MG, Bernardi S, Puro V, et al. Percentage and determinants of missed HIV testing in pregnancy: a survey of women delivering in the Lazio region, Italy. AIDS care. 2014;26(7):899-906.
28. Barigye H, Levin J, Maher D, Tindiwegi G, Atuhumuza E, Nakibinge S, et al. Operational evaluation of a service for prevention of mother-to-child transmission of HIV in rural Uganda: barriers to uptake of single-dose nevirapine and the role of birth reporting. Tropical medicine & international health. 2010;15(10):1163-71.
29. Malaju MT, Alene GD. Assessment of utilization of provider-initiated HIV testing and counseling as an intervention for prevention of mother to child transmission of HIV and associated factors among pregnant women in Gondar town, North West Ethiopia. BMC Public Health. 2012;12:226.
30. Vieira N, Rasmussen DN, Oliveira I, Gomes A, Aaby P, Wejse C, et al. Awareness, attitudes and perceptions regarding HIV and PMTCT amongst pregnant women in Guinea-Bissau- a qualitative study. BMC women's health. 2017;17(1):71.

31. Liao C, Golden WC, Anderson JR, Coleman JS. Missed opportunities for repeat HIV testing in pregnancy: implications for elimination of mother-to-child transmission in the United States. AIDS Patient Care STDS. 2017;31(1):20-6.
32. Gebremedhin T, Alamneh TS, Hagos A, Desalegn B, Worku N. A multilevel analysis of determinants of PMTCT service utilisation among women during the antepartum, intrapartum and postpartum period in Ethiopia. BMC Pregnancy Childbirth. 2021;21(1):1-10.
33. Madhivanan P, Krupp K, Kulkarni V, Kulkarni S, Vaidya N, Shaheen R, et al. HIV testing among pregnant women living with HIV in India: are private healthcare providers routinely violating women's human rights? BMC international health and human rights. 2014;14(1):1-9.
34. Seidu A-A, Oduro JK, Ahinkorah BO, Budu E, Appiah F, Baatiema L, et al. Women's healthcare decision-making capacity and HIV testing in sub-Saharan Africa: a multi-country analysis of demographic and health surveys. BMC Public Health. 2020;20(1):1-11.
35. Ford CE, Coetzee D, Winston J, Chibwesha CJ, Ekouevi DK, Welty TK, et al. Maternal decision-making and uptake of health services for the prevention of mother-to-child HIV transmission: a secondary analysis. Matern Child Health J. 2019;23(1):30-8.
36. Stinson K, Myer L. Barriers to initiating antiretroviral therapy during pregnancy: a qualitative study of women attending services in Cape Town, South Africa. African Journal of AIDS research. 2012;11(1):65-73.
37. Abtew S, Awoke W, Asrat A. Acceptability of provider-initiated HIV testing as an intervention for prevention of mother to child transmission of HIV and associated factors among pregnant women attending at Public Health Facilities in Assosa town, Northwest Ethiopia. BMC research notes. 2015;8:661.
38. Kohler PK, Ondenge K, Mills LA, Okanda J, Kinuthia J, Olilo G, et al. Shame, guilt, and stress: Community perceptions of barriers to engaging in prevention of mother to child transmission (PMTCT) programs in western Kenya. AIDS Patient Care STDS. 2014;28(12):643-51.
39. Mustapha M, Musiime V, Bakeera-Kitaka S, Rujumba J, Nabukeera-Barungi N. Utilization of "prevention of mother-to-child transmission" of HIV services by adolescent and young mothers in Mulago Hospital, Uganda. BMC infectious diseases. 2018;18(1):1-11.
40. Cook RE, Ciampa PJ, Sidat M, Blevins M, Burlison J, Davidson MA, et al. Predictors of successful early infant diagnosis of HIV in a rural district hospital in Zambezia, Mozambique. Journal of acquired immune deficiency syndromes (1999). 2011;56(4):e104.
41. Ankrah AK, Dako-Gyeke P. Factors influencing the delivery and uptake of early infant diagnosis of HIV services in Greater Accra, Ghana: A qualitative study. PLoS One. 2021;16(2):e0246876.
42. Mofenson LM, Cohn J, Sacks E. Challenges in the Early Infant HIV Diagnosis and Treatment Cascade. JAIDS Journal of Acquired Immune Deficiency Syndromes. 2020;84:S1-S4.
43. Adeniyi VO, Thomson E, Goon DT, Ajayi IA. Disclosure, stigma of HIV positive child and access to early infant diagnosis in the rural communities of OR Tambo District, South Africa: a qualitative exploration of maternal perspective. BMC pediatrics. 2015;15(1):1-10.
44. Shargie MB, Eek F, Abaychew A. Prophylactic treatment uptake and compliance with recommended follow up among HIV exposed infants: a retrospective study in Addis Ababa, Ethiopia. BMC research notes. 2011;4(1):1-8.
45. Kebede B, Gebeyehu A, Jain S, Sun S, Haubrich R. Delay in early infant diagnosis and high loss to follow-up among infant born to HIV-infected women in Ethiopia. World Journal of AIDS. 2014;4(04):402.
46. Moher D, Liberati A, Tetzlaff J, Altman DG, Group P. Preferred reporting items for systematic reviews and meta-analyses: the PRISMA statement. Int J Surg. 2010;8(5):336-41.
47. United Nations Department of Economic and social afairs statistical division. Standard country or area codes for statistical use 2020 [cited 2020 6 December]. Available from: https://unstats.un.org/unsd/methodology/m49/

48. Impact of an innovative approach to prevent mother-to-child transmission of HIV--Malawi, July 2011-September 2012. MMWR Morb Mortal Wkly Rep. 2013;62(8):148-51.
49. Pace R, Pluye P, Bartlett G, Macaulay AC, Salsberg J, Jagosh J, et al. Testing the reliability and efficiency of the pilot Mixed Methods Appraisal Tool (MMAT) for systematic mixed studies review. Int J Nurs Stud. 2012;49(1):47-53.
50. Allen B, Canuto K, Evans JR, Lewis E, Gwynn J, Radford K, et al. Facilitators and Barriers to Physical Activity and Sport Participation Experienced by Aboriginal and Torres Strait Islander Adults: A Mixed Method Review. Int J Environ Res Public Health. 2021;18(18).
51. Laher Z, Robertson N, Harrad-Hyde F, Jones CR. Prevalence, Predictors, and Experience of Moral Suffering in Nursing and Care Home Staff during the COVID-19 Pandemic: A Mixed-Methods Systematic Review. Int J Environ Res Public Health. 2022;19(15):9593.
52. Mey A, Plummer D, Dukie S, Rogers GD, O'Sullivan M, Domberelli A. Motivations and barriers to treatment uptake and adherence among people living with HIV in Australia: a mixed-methods systematic review. Aids Behav. 2017;21(2):352-85.
53. Thorlund K, Imberger G, Johnston BC, Walsh M, Awad T, Thabane L, et al. Evolution of heterogeneity (I2) estimates and their 95% confidence intervals in large meta-analyses. PLoS One. 2012;7(7):e39471.
54. Stern C, Lizarondo L, Carrier J, Godfrey C, Rieger K, Salmond S, et al. Methodological guidance for the conduct of mixed methods systematic reviews. JBI Evid Implement. 2021;19(2):120-9.
55. Andersen RM. Revisiting the behavioral model and access to medical care: does it matter? J Health Soc Behav. 1995;36(1):1-10.
56. Astawesegn FH, Stulz V, Agho KE, Mannan H, Conroy E, Ogbo FA. Prenatal HIV Test Uptake and Its Associated Factors for Prevention of Mother to Child Transmission of HIV in East Africa. Int J Environ Res Public Health. 2021;18(10).
57. Abtew S, Awoke W, Asrat A. Acceptability of provider-initiated HIV testing as an intervention for prevention of mother to child transmission of HIV and associated factors among pregnant women attending at Public Health Facilities in Assosa town, Northwest Ethiopia. BMC research notes. 2015;8(1):1-8.
58. Akal CG, Afework DT. Status of Prevention of Mother-to-Child Transmission (PMTCT) Services Utilization and Factors Affecting PMTCT Service Uptake by Pregnant Women Attending Antenatal Care Clinic in Selected Health Facilities of Afar Regional State, Ethiopia. J Environ Public Health. 2018;2018 (no pagination).
59. Berhan Z, Abebe F, Gedefaw M, Tesfa M, Assefa M, Tafere Y. Risk of HIV and associated factors among infants born to HIV positive women in Amhara region, Ethiopia: a facility based retrospective study. BMC research notes. 2014;7:876.
60. Desta ML, Saravanan M, Hilekiros H, Kahsay AG, Mohamed NF, Gezahegn AA, et al. HIV prevalence and risk factors in infants born to HIV positive mothers, measured by dried blood spot real-time PCR assay in Tigray, Northern Ethiopia. BMC pediatrics. 2019;19(1):1-8.
61. Ebuy H, Bekele A, Redae G. HIV testing, test results and factors influencing among infants born to HIV positive mothers in public hospitals of Mekelle City, North Ethiopia: a cross-sectional study. BMC Infectious Diseases. 2020;20(1):1-10.
62. Ejigu Y, Tadesse B. HIV testing during pregnancy for prevention of mother-to-child transmission of HIV in Ethiopia. PLoS One. 2018;13(8):e0201886.
63. Gebeyehu NA, Wassie AY, Gelaw KA. Acceptance of HIV testing and associated factors among pregnant women attending antenatal care in Gunino health center, Southern Ethiopia 2019: an institutional based cross-sectional study. HIV/AIDS (Auckland, NZ). 2019;11:333.
64. Gebremedhin KB, Tian B, Tang C, Zhang X, Yisma E, Wang H. Factors associated with acceptance of provider-initiated HIV testing and counseling among pregnant women in Ethiopia. Patient preference and adherence. 2018;12:183.

65. Gebresillassie BM, Emiru YK, Erku DA, Mersha AG, Mekuria AB, Ayele AA, et al. Utilization of Provider-Initiated HIV Testing and Counseling as an Intervention for PMTCT Services Among Pregnant Women Attending Antenatal Clinic in a Teaching Hospital in Ethiopia. Front Public Health. 2019;7.
66. Moges NA, Kassa GM, Boneya DJ. Rate of HIV transmission and associated factors among HIV-exposed infants in selected health facilities of East and West Gojjam Zones, Northwest Ethiopia; retrospective cohort study. BMC Infectious Diseases. 2017;17:1-10.
67. Tadewos K, Adimasu M, Tachbele E. Mother-to-Child Transmission of HIV and Associated Factors Among Exposed Infants in Pastoralist Health Facilities, South Omo Zone, Ethiopia, 2020–A Retrospective Cross-Sectional Study. HIV/AIDS (Auckland, NZ). 2021;13:1015.
68. Tsehay AK. Factors associated with HIV-positive sero-status among exposed infants attending care at health facilities in Bahir Dar administration, Ethiopia: Evidence from medical records. Cogent Medicine. 2019;6(1) (no pagination).
69. Workagegn F, Kiros G, Abebe L. Predictors of HIV-test utilization in PMTCT among antenatal care attendees in government health centers: Institution-based cross-sectional study using health belief model in Addis Ababa, Ethiopia, 2013. HIV/AIDS - Research and Palliative Care. 2015;7:215-22.
70. Wudineh F, Damtew B. Mother-to-child transmission of HIV infection and its determinants among exposed infants on care and follow-up in Dire Dawa City, Eastern Ethiopia. AIDS research and treatment. 2016;2016.
71. Zegeye B, Lemma G, Balcha A, Taderegew MM. Prevention of Mother-to-Child HIV Transmission Service Utilization among Pregnant Women in Northeast Ethiopia: A Cross-Sectional Survey. Scientifica. 2020;2020.
72. Olana T, Bacha T, Worku W, Tadesse BT. Early infant diagnosis of HIV infection using DNA-PCR at a referral center: an 8 years retrospective analysis. AIDS research and therapy. 2016;13(1):1-7.
73. Deressa W, Seme A, Asefa A, Teshome G, Enqusellassie F. Utilization of PMTCT services and associated factors among pregnant women attending antenatal clinics in Addis Ababa, Ethiopia. BMC Pregnancy Childbirth. 2014;14(1):1-13.
74. Gaitho D, Kinoti F, Mwaniki L, Kemunto D, Ogoti V, Njigua C, et al. Factors associated with the timely uptake of initial HIV virologic test among HIV-exposed infants attending clinics within a faith-based HIV program in Kenya; a cross-sectional study. BMC Public Health. 2021;21(1):1-7.
75. Haider MR, Kingori C, Gebre HS. Factors associated with HIV testing among women during pregnancy in Kenya: evidence from the Kenya Demographic and Health Survey 2014. AIDS Care. 2022;34(2):193-200.
76. Makau G, Okwara F, Oyore J. Determinants of early infant diagnosis and treatment of HIV among exposed infants in informal settlements in Nairobi, Kenya. East Cent Afr Med J. 2015;2:74-9.
77. Ongaki D, Obonyo M, Nyanga N, Ransom J. Factors Affecting Uptake of PMTCT Services, Lodwar County Referral Hospital, Turkana County, Kenya, 2015 to 2016. Journal of the International Association of Providers of AIDS Care. 2019;18(no pagination).
78. Bobrow EA, Yemaneberhan AG, Phiri M, Katirayi L, Ahimbisibwe A, Chimbwandira F, et al. Barriers, facilitators and recommendations for the early infant diagnosis and treatment (EIDT) cascade: A qualitative study in Malawi. SAJCH South African Journal of Child Health. 2016;10(2):116-20.
79. Cataldo F, Chiwaula L, Nkhata M, van Lettow M, Kasende F, Rosenberg NE, et al. Exploring the experiences of women and health care workers in the context of PMTCT Option B Plus in Malawi. Journal of acquired immune deficiency syndromes (1999). 2017;74(5):517.
80. Ng'ambi WF, Merzouki FA, Estill J, Orel E, Chimpandule T, Nyirenda R, et al. Factors associated with the risk of HIV infection among HIV-exposed infants in Malawi: 2013–2020. BMJ paediatrics open. 2022;6(1).

81. Ahoua L, Tiendrebeogo T, Arikawa S, Lahuerta M, Aly D, Journot V, et al. PMTCT care cascade and factors associated with attrition in the first four years after Option B+ implementation in Mozambique. Tropical Medicine & International Health. 2020;25(2):222-35.
82. Buleza Lamucene O, Bernales M, Irarrázabal Vargas L, Ferrer Lagunas L. Perceptions of barriers and facilitators to implement programs for prevention of mother-to-child transmission of HIV-Mozambique. Revista da Escola de Enfermagem da USP. 2022;56.
83. Lain MG, Chicumbe S, de Araujo AR, Karajeanes E, Couto A, Giaquinto C, et al. Correlates of loss to follow-up and missed diagnosis among HIV-exposed infants throughout the breastfeeding period in southern Mozambique. PLoS One. 2020;15(8):e0237993.
84. Thidor GTY, August F. Prevention of mother-to-child transmission of HIV: knowledge, attitudes and practice among pregnant women at Juba Teaching Hospital. South Sudan med j. 2019;12(1):12-6.
85. Bwana VM, Mfinanga SG, Simulundu E, Mboera LE, Michelo C. Accessibility of early infant diagnostic services by under-5 years and HIV exposed children in Muheza District, north-East Tanzania. Front Public Health. 2018;6:139.
86. Gamell A, Luwanda LB, Kalinjuma AV, Samson L, Ntamatungiro AJ, Weisser M, et al. Prevention of mother-to-child transmission of HIV Option B+ cascade in rural Tanzania: The One Stop Clinic model. PLoS One. 2017;12(7) (no pagination).
87. Konje ET, Magoma MTN, Hatfield J, Kuhn S, Sauve RS, Dewey DM. Missed opportunities in antenatal care for improving the health of pregnant women and newborns in Geita district, Northwest Tanzania. BMC Pregnancy Childbirth. 2018;18(1):1-13.
88. Nungu SI, Mghamba JM, Rumisha SF, Semali IA. Uptake and determinants for HIV postpartum re-testing among mothers with prenatal negative status in Njombe region, Tanzania. BMC Infectious Diseases. 2019;19(1) (no pagination).
89. Oshosen M, Knettel BA, Knippler E, Relf M, Mmbaga BT, Watt MH. "She just told me not to cry": A qualitative study of experiences of HIV Testing and Counseling (HTC) among pregnant women living with HIV in Tanzania. Aids Behav. 2021;25(1):104-12.
90. Bergmann J, Wanyenze R, Makumbi F, Naigino R, Kiene S, Stockman J. Maternal Influences on Access to and Use of Infant ARVs and HIV Health Services in Uganda. AIDS & Behavior. 2017;21(9):2693-702.
91. Buregyeya E, Naigino R, Mukose A, Makumbi F, Esiru G, Arinaitwe J, et al. Facilitators and barriers to uptake and adherence to lifelong antiretroviral therapy among HIV infected pregnant women in Uganda: a qualitative study. BMC Pregnancy & Childbirth. 2017;17:1-9.
92. Mukose AD, Bastiaens H, Makumbi F, Buregyeya E, Naigino R, Musinguzi J, et al. What influences uptake and early adherence to Option B+ (lifelong antiretroviral therapy among HIV positive pregnant and breastfeeding women) in Central Uganda? A mixed methods study. PLoS ONE. 2021;16(5 May) (no pagination).
93. Hampanda KM, Nimz AM, Abuogi LL. Barriers to uptake of early infant HIV testing in Zambia: the role of intimate partner violence and HIV status disclosure within couples. AIDS research and therapy. 2017;14(1):1-9.
94. Kanguya T, Koyuncu A, Sharma A, Kusanathan T, Mubanga M, Chi BH, et al. Identifying barriers to ART initiation and adherence: An exploratory qualitative study on PMTCT in Zambia. PLoS ONE. 2022;17(1 January 2022) (no pagination).
95. Augustine N, Philip O, Kumar AM, Simukai Z, Owen M, Dumisani MH, et al. Gaps in the care cascade among human immunodeficiency virus-exposed infants born in 2017 in Mashonaland East Province of Zimbabwe. Journal of Global Infectious Diseases. 2021;13(2):72.
96. Dzangare J, Takarinda KC, Harries AD, Tayler-Smith K, Mhangara M, Apollo TM, et al. HIV testing uptake and retention in care of HIV-infected pregnant and breastfeeding women initiated on 'Option B+' in rural Zimbabwe. Tropical Medicine and International Health. 2016;21(2):202-9.
97. Wanyenze RK, Goggin K, Finocchario-Kessler S, Beyeza-Kashesya J, Mindry D, Birungi J, et al. Utilization of prevention of mother-to-child transmission (PMTCT) services among pregnant women

in HIV care in Uganda: a 24-month cohort of women from pre-conception to post-delivery. BMC research notes. 2018;11(1):187.
98. Chadambuka A, Katirayi L, Muchedzi A, Tumbare E, Musarandega R, Mahomva AI, et al. Acceptability of lifelong treatment among HIV-positive pregnant and breastfeeding women (Option B+) in selected health facilities in Zimbabwe: a qualitative study. BMC Public Health. 2018;18(1):1-8.
99. Kim MH, Zhou A, Mazenga A, Ahmed S, Markham C, Zomba G, et al. Why did I stop? Barriers and facilitators to uptake and adherence to ART in Option B+ HIV care in Lilongwe, Malawi. PLoS One. 2016;11(2):e0149527.
100. Semali I, Damian DJ, Saronga HP, Malamsha D. Factors associated with HIV testing and receiving results during antenatal care in Tanzania. African Population Studies. 2014:1035-45.
101. Ford C, Chibwesha CJ, Winston J, Jacobs C, Lubeya MK, Musonda P, et al. Women's decision-making and uptake of services to prevent mother-to-child HIV transmission in Zambia. AIDS care. 2018;30(4):426-34.
102. Yaya S, Oladimeji O, Oladimeji KE, Bishwajit G. Determinants of prenatal care use and HIV testing during pregnancy: a population-based, cross-sectional study of 7080 women of reproductive age in Mozambique. BMC Pregnancy Childbirth. 2019;19(1):1-10.
103. McCoy SI, Buzdugan R, Padian NS, Musarandega R, Engelsman B, Martz TE, et al. Uptake of services and behaviors in the prevention of mother-to-child HIV transmission (PMTCT) cascade in Zimbabwe. Journal of acquired immune deficiency syndromes (1999). 2015;69(2):e74.
104. Adebimpe WO. Challenges facing early infant diagnosis of HIV among infants in resource poor settings. African J Reprod Health. 2013;17(1):122-9.
105. United Nations Programme on HIV/AIDS. Policy statement: UNAIDS/WHO POLICY STATEMENT ON HIV TESTING: UNAIDS; 2004 [Available from: http://data.unaids.org/una-docs/hivtestingpolicy_en.pdf.
106. Open Society Initiative For East Africa. HIV/AIDS, Human Rights, and Legal Services in Uganda: A Country Assessment

2008 [cited 2021 22 February]. Available from: https://www.refworld.org/pdfid/4cdcead32.pdf.
107. Kasenga F, Byass P, Emmelin M, Hurtig AK. The implications of policy changes on the uptake of a PMTCT programme in rural Malawi: first three years of experience. Glob Health Action. 2009;2.
108. Gunn JK, Asaolu IO, Center KE, Gibson SJ, Wightman P, Ezeanolue EE, et al. Antenatal care and uptake of HIV testing among pregnant women in sub-Saharan Africa: a cross-sectional study. J Int Aids Soc. 2016;19(1):20605.
109. Creek T, Ntumy R, Mazhani L, Moore J, Smith M, Han G, et al. Factors associated with low early uptake of a national program to prevent mother to child transmission of HIV (PMTCT): results of a survey of mothers and providers, Botswana, 2003. Aids Behav. 2009;13(2):356-64.
110. Rennie S, Behets F. Desperately seeking targets: the ethics of routine HIV testing in low-income countries. B World Health Organ. 2006;84:52-7.
111. Castro A, Farmer P. Understanding and addressing AIDS-related stigma: from anthropological theory to clinical practice in Haiti. Am J Public Health. 2005;95(1):53-9.
112. Mohammadi F, Kohan S, Mostafavi F, Gholami A. The stigma of reproductive health services utilization by unmarried women. Iranian Red Crescent Medical Journal. 2016;18(3).
113. Newton-Levinson A, Winskell K, Abdela B, Rubardt M, Stephenson R. 'People insult her as a sexy woman': sexuality, stigma and vulnerability among widowed and divorced women in Oromiya, Ethiopia. Culture, health & sexuality. 2014;16(8):916-30.
114. Bekele YA, Fekadu GA. Factors associated with HIV testing among young females; further analysis of the 2016 Ethiopian demographic and health survey data. PLoS One. 2020;15(2):e0228783.
115. Novignon J, Ofori B, Tabiri KG, Pulok MH. Socioeconomic inequalities in maternal health care utilization in Ghana. Int J Equity Health. 2019;18(1):141.

116. Makinen M, Waters H, Rauch M, Almagambetova N, Bitran R, Gilson L, et al. Inequalities in health care use and expenditures: empirical data from eight developing countries and countries in transition. B World Health Organ. 2000;78(1):55-65.
117. Alam N, Hajizadeh M, Dumont A, Fournier P. Inequalities in maternal health care utilization in sub-Saharan African countries: a multiyear and multi-country analysis. PLoS One. 2015;10(4):e0120922.
118. Goli S, Nawal D, Rammohan A, Sekher TV, Singh D. DECOMPOSING THE SOCIOECONOMIC INEQUALITY IN UTILIZATION OF MATERNAL HEALTH CARE SERVICES IN SELECTED COUNTRIES OF SOUTH ASIA AND SUB-SAHARAN AFRICA. Journal of biosocial science. 2018;50(6):749-69.
119. Awopegba OE, Kalu A, Ahinkorah BO, Seidu A-A, Ajayi AI. Prenatal care coverage and correlates of HIV testing in sub-Saharan Africa: Insight from demographic and health surveys of 16 countries. PLoS One. 2020;15(11):e0242001.
120. Sambah F, Baatiema L, Appiah F, Ameyaw EK, Budu E, Ahinkorah BO, et al. Educational attainment and HIV testing and counselling service utilisation during antenatal care in Ghana: analysis of demographic and health surveys. PLoS One. 2020;15(1):e0227576.
121. Rao A, Schwartz S, Billong SC, Bowring A, Fouda G, Ndonko F, et al. Predictors of early childhood HIV testing among children of sex workers living with HIV in Cameroon. BMC Public Health. 2019;19(1):1-8.
122. Munkhondya TEM, Smyth RM, Lavender T. Facilitators and barriers to retention in care under universal antiretroviral therapy (Option B+) for the Prevention of Mother to Child Transmission of HIV (PMTCT): A narrative review. International Journal of Africa Nursing Sciences. 2021;15:100372.

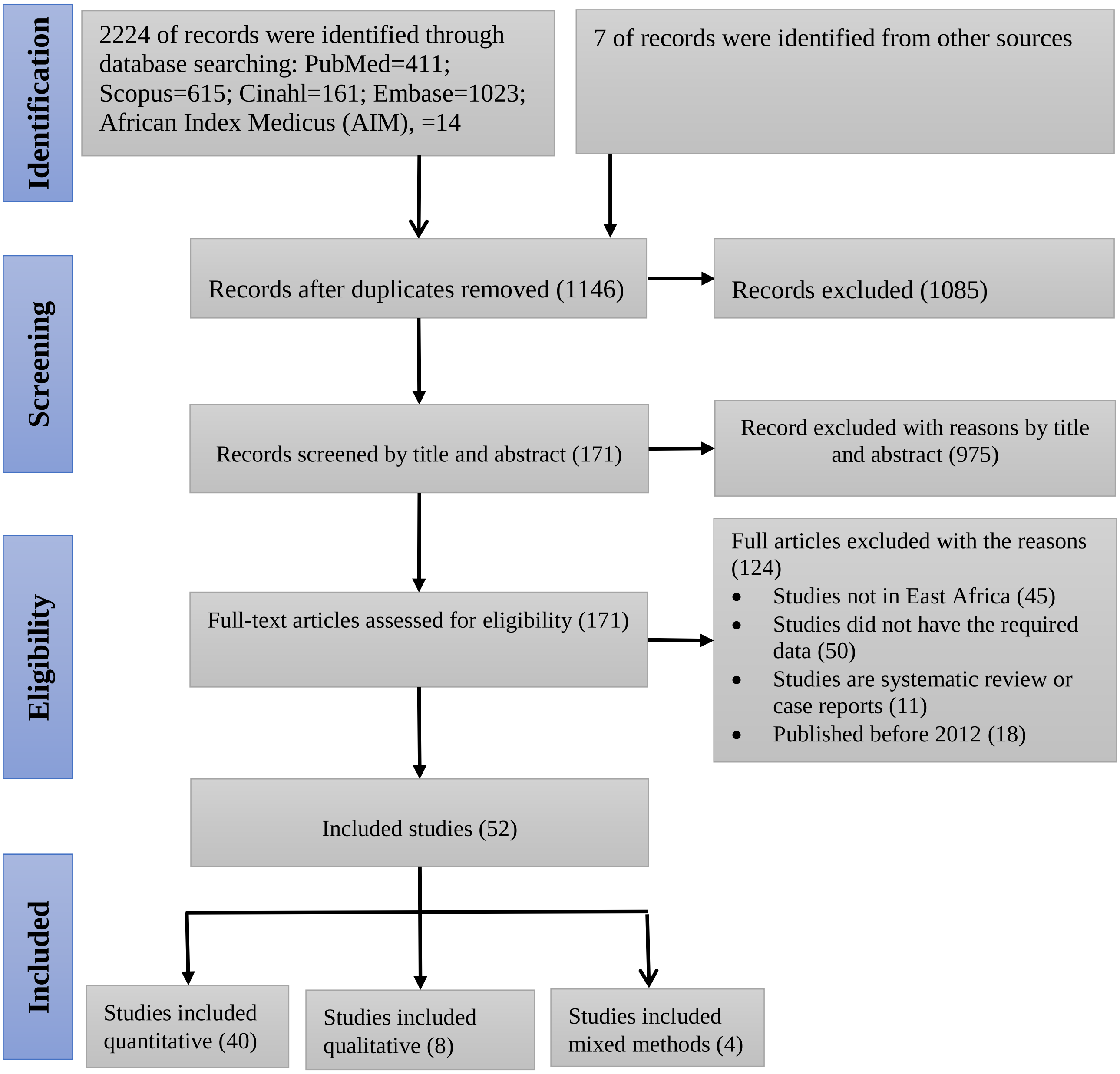

Figure 1: PRISMA flow chart

S5 Figure: Funnel plot to assess publication bias A) Maternal HIV testing, B) Maternal ART uptake, C) Infant ARV prophylaxis, D) Infant ARV prophylaxis in East Africa

Figure

Click here to access/download;Figure;Figure2a.tif

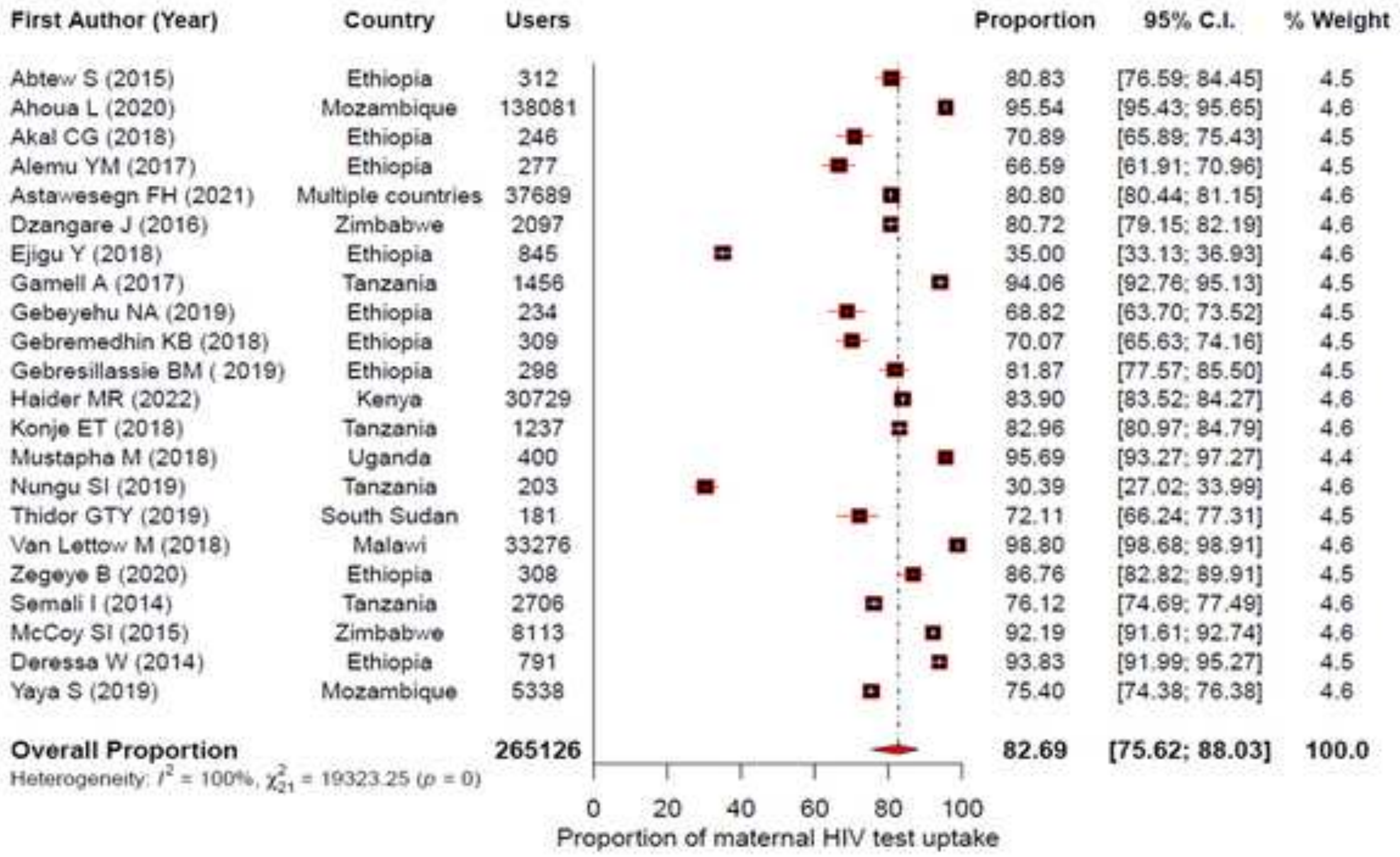

Figure

Click here to access/download;Figure;Figure2b.tif

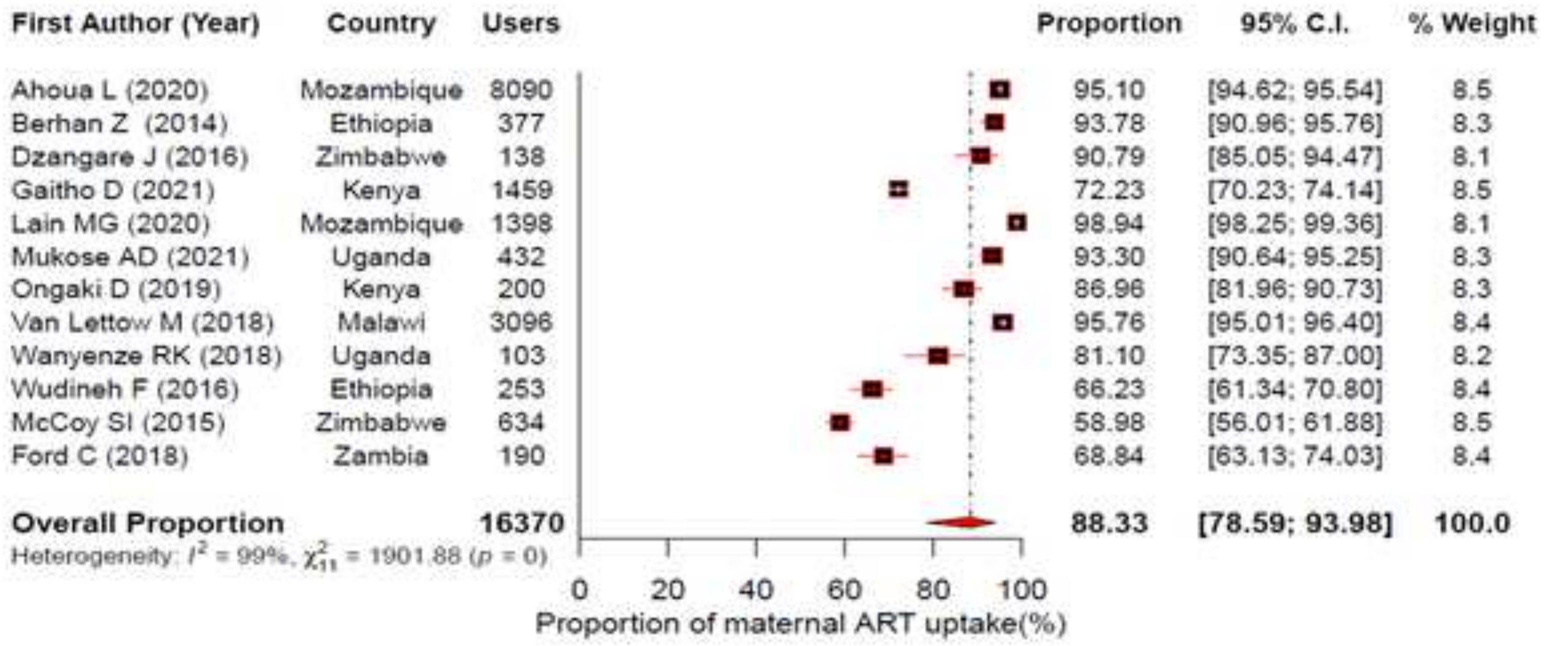

Figure

Click here to access/download;Figure;Figure2c.tif

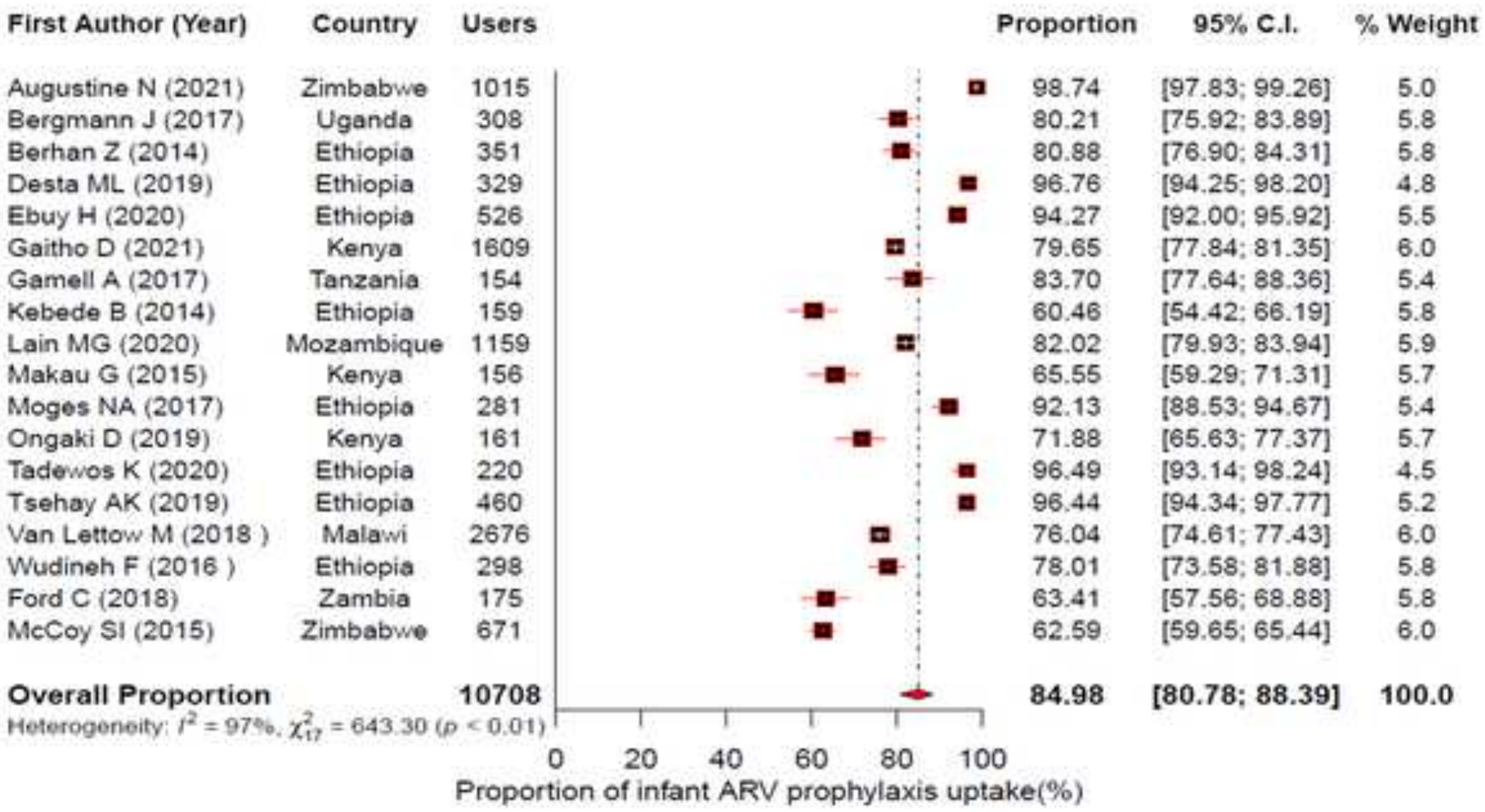

Figure
Click here to access/download;Figure;Figure2d.tif

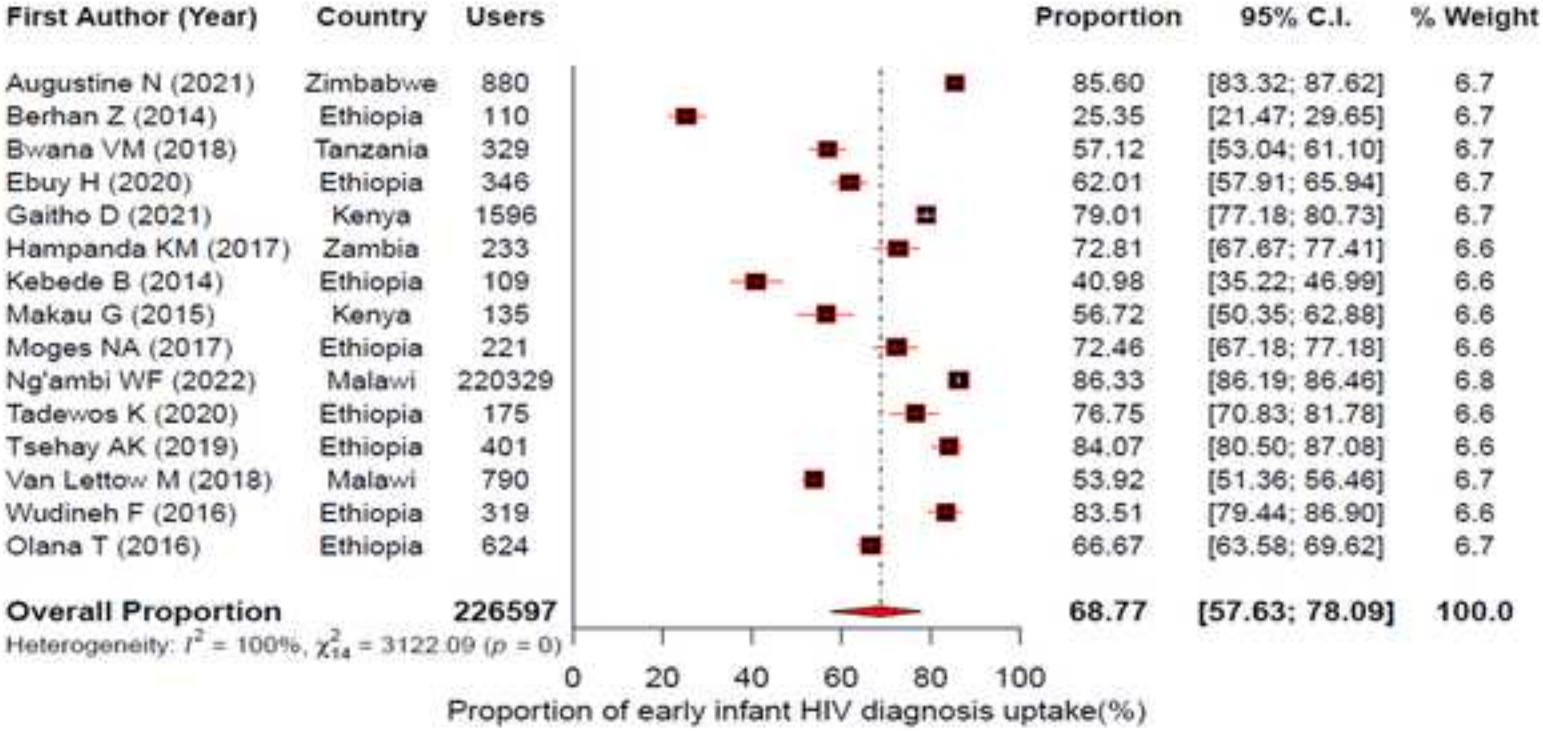

Click here to access/download
Supporting Information
S1 Table. PRISMA 2009 Checklist.docx

Click here to access/download
Supporting Information
S2 Table. Search strategy.doc

Click here to access/download
Supporting Information
S3 Table. Methodological quality assessment.docx

Click here to access/download
Supporting Information
S1 Figure. Publication bias.tif

Click here to access/download
Supporting Information
S4 Table. Subgroup analysis..docx
