# Supplementary material for: Understanding the uptake and determinants of prevention of mother-to-child transmission of HIV services in East Africa: Mixed methods systematic review and meta-analysis
Source: PLoS One. 2024 Apr 18;19(4):e0300606. doi: 10.1371/journal.pone.0300606 (PMC11025786; doi:10.1371/journal.pone.0300606)
Supplement: Attachment — Submitted filename: Response to Reviewers.docx [file pone.0300606.s007.docx]

**Date: 06/08/2023**

**Title:** **Understanding** **the uptake and determinants of prevention of mother-to-child transmission of HIV services in East Africa: Mixed methods systematic review and meta-analysis**

**Dear Editor and Reviewers:** We really thank you for your valuable comments/suggestions for the betterment of our manuscript. Your concerns and suggestions are addressed in the revised manuscript and below we have tried to clarify some of the points raised by the reviewers.

**Reviewer # 1**

1. The paper would benefit from a time-stratified analysis, differentiating between HIV service delivery approaches before and after the World Health Organization's "Treat All" guidelines in 2016. Access to HIV testing and treatment dramatically advanced with this guidance, with many women receiving HIV diagnosis and treatment before pregnancy which is known to be superior to diagnosis and initiation of treatment during pregnancy from a PMTCT perspective. As presented, the analysis compiles findings from studies that were conducted in dramatically different HIV service delivery contexts and may not present the most relevant findings for programs.

**Author response:** As you mentioned, there has been a significant shift in the HIV guidelines, such as moving away from considering WHO stage or CD4 count levels to adopting a test and treat approach for the general population in 2016`. However, when it comes to PMTCT, the approach has evolved differently due to its specific goal of preventing vertical transmission.

Over the last two decades, WHO has developed various PMTCT guidelines/approaches, such as option A, option B and option B+. Option B+ involves a test and treat approach for HIV-positive pregnant women only with the primary objective of preventing vertical transmission from mother to child and has been in effect since 2012 (Malawi was the first country to implement this in East Africa). Therefore, given the focus of this review on understanding the current status of preventing mother-to-child HIV transmission, articles published after the implementation of Option B+ (since 2012) were included in this review to gather relevant information.

**Reviewer # 2**

2. To my understanding Ethiopia, Mozambique, Zimbabwe, etc are not part of East Africa. I suggest that authors should review or justify the inclusion of these countries.

**Author response:** Regarding the country's classification, there are different classifications of East African countries. In this study, we selected the United Nations Statistical Division and World Health Organization classification of East Africa region as mostly health-related reports and studies considered this classification. We have cited the following reference to indicate the list of East African countries in the manuscript method section (see **page 5 lines 128-129**).

- *United Nations Department of Economic and social affairs statistical division. Standard country or area codes for statistical use 2020 [Available from:* [*https://unstats.un.org/unsd/methodology/m49/*](https://unstats.un.org/unsd/methodology/m49/)
- United Nation. World Population Review 2020 [Available from: <https://worldpopulationreview.com/country-rankings/east-african-countries>

Furthermore, recently published studies (including those published by Plos One) using the same countries under East Africa are cited below for the reviewer's consideration:

1. *Tesema GA, Tessema ZT. Pooled prevalence and associated factors of health facility delivery in East Africa: mixed-effect logistic regression analysis. PLoS One. 2021 Apr 23;16(4):e0250447.*
2. *Raru TB, Ayana GM, Zakaria HF, Merga BT. Association of higher educational attainment on antenatal care utilization among pregnant women in East Africa using Demographic and Health Surveys (DHS) from 2010 to 2018: a multilevel analysis. International journal of women's health. 2022 Feb 1:67-77.*
3. From the review findings, the authors can suggest specific strategies/implemented target interventions.

**Author response:** Thank you so much. We have included specific strategies/implemented target interventions as part of the discussion and conclusion ( see **page 30 lines 537-541)**

4. Was the protocol of this systematic review registered? Authors should provide information.

**Author response:** We were unable to register the protocol because of the time limitations we encountered. During that time, we encountered difficulty in registering a protocol on platforms such as PROSPERO, due to the vast amount of reviews related to COVID-19 that had overloaded them.

5. Authors should clarify the meta-analyses mentioned here in relation to the following statement: "...it was not feasible to conduct a meta-analysis to assess the effect of each factor."

**Author response**: Thank you so much. To clarify this, a meta-analysis was done to estimate PMTCT services uptake in percentage, as we have indicated under the method section **(page 7 lines 198-199).** The result of the meta-analysis for the service uptake has been presented on **page 14**. Whereas, for the determinants or factors associated with PMTCT service uptake, we did not conduct a meta-analysis because of wide variations in the measurement and classification of the variables between studies **(see** **pages 7 line number 191-194**). The result of narrative synthesis for the factor has been presented on **pages 15-26**

6. General comment: In comparison with previous systematic reviews on the same topic, what are new findings that this review has found different from those that have filled the existing knowledge gap?

**Author response:** Unlike other reviews, this review holistically addressed the uptake and determinants of key PMTCT services cascade such as (i) maternal HIV testing among pregnant/postpartum women, (ii) ART initiation among HIV-infected pregnant/postpartum women, (iii) initiation of ARV prophylaxis for HIV exposed infants (HEIs), and (iv) early infant diagnosis (EID)/HIV test at six weeks of age/ in East Africa.  It enables readers to compare uptake and determinants across the cascade.  Most previous systematic reviews included only quantitative studies, whereas this review included studies that employed mixed methods (quantitative and qualitative). after the implementation of option B+. Most previous studies done after the implementation of option b+ were focused on determinants of ART adherence, however, this review focuses on determinants of PMTCT cascade uptake

7. Can authors justify, the reasons for not searching for grey literature, and if it has affected their review findings?

**Author response**: To focus on peer-reviewed and published articles.

8. What about limited number of HCWs??

**Author response:** We have provided suggestions to improve the number of HCWs under the conclusion section **( page 30, lines 540-541)**

**Reviewer #3**

9. Authors should provide the confidence intervals of the Adjusted Odd Ratios (AOR).

**Author response:** Thank you, we have addressed this omission in the revised manuscript table 2 **(pages 17-21).**

We appreciate the editor and reviewers for the comments and time taken to read our manuscript. And hope that we have addressed the reviewers’ comments and associated changes in the manuscript to your satisfaction.

Thank you.

Feleke Hailemichael
