# Supplementary material for: Understanding the uptake and determinants of prevention of mother-to-child transmission of HIV services in East Africa: Mixed methods systematic review and meta-analysis
Source: PLoS One. 2024 Apr 18;19(4):e0300606. doi: 10.1371/journal.pone.0300606 (PMC11025786; doi:10.1371/journal.pone.0300606)
Supplement: Attachment — Submitted filename: Response to Reviewers.docx [file pone.0300606.s008.docx]

**Date: 12/02/2024**

**Title:** **Understanding** **the uptake and determinants of prevention of mother-to-child transmission of HIV services in East Africa: Mixed methods systematic review and meta-analysis**

**Dear Editor and Reviewers:** We really thank you for your valuable comments/suggestions for the improvement of our manuscript. Your concerns and suggestions are addressed in the revised manuscript and below we have tried to clarify some of the points raised by the reviewers.

**Reviewers comment**

**Reviewers**: The high variability of the studies included in this meta-analysis makes it difficult to summarize the results and recommendations. Undoubtedly the work was exhaustive, however: despite the detailed description of the objective of the studies considered for the quantitative pole (column 2 Table 1),

1/the main measurement(s) for each of the studies does not appear in Table 1. Several selected studies focus on antenatal screening, antenatal monitoring, and early diagnosis; proportionally fewer studies target antiretroviral treatment during pregnancy or afterwards. 2/Classifying the studies in Table 1 according to the target of the PMTCT cascade could be recommended. if possible

**Authors**: Thank you for your comment. I have included targeted PMTCT cascades in Table 1 column 7 with footnote description.

**Reviewers**: Within the limits of the study, in addition to those cited, the authors could mention the failure to take into account in their cascade the time of final diagnosis at 18 months of children exposed to HIV; Because it is the central indicator for the elimination of PMTCT as well as they could mention not having taken into account the questions of adherence to antiretroviral treatment of pregnant/lactating women and the retention of mother babies couples in follow-up up to 18 -24 months ( the end of PMTCT CASCADE)

**Authors**: We appreciate the reviewer's concern. Of course, in places where there is no virological testing, babies typically must wait until they are 18-24 months old to undergo an antigen-antibody test. This is because it's expected that maternal antigen will clear out of the infant's blood by 18-24 months, allowing for accurate antigen tests. However, this review focused on the recommended "early" infant diagnosis, which involves testing HIV-exposed infants within 6 weeks of birth. Early infant diagnosis employs a virological test using DNA-PCR to directly detect HIV. This suggestion is advised to prevent infant deaths, as infants with HIV have a high likelihood of death because of the disease before reaching the age of two. It is advised for countries to establish virological testing using HIV-DNA (PCR) and/or HIV-RNA at least at central or tertiary levels to enable early HIV testing for infants. Therefore, the study, as outlined in the methods section, aims to examine the uptake and determinants of "early infant diagnosis”, involving virological tests before 6 weeks.

**Reviewers**: In the conclusion and recommendations line 540 541, while mentioning expert mothers, authors should reinforce their references with the experience of mothers 2 mothers. (As this is not coming from the findings of their review).

**Response**: As you mentioned, involving expert mothers while dealing with one mother to another was not the finding of the review, we rather recommended involving expert mothers as a solution for the staff shortage we identified in this review. Expert mothers (EMs) are mothers living with HIV who have personal experience with PMTCT programs, which they can draw upon to support other women and their families. Previous studies showed that the involvement of expert mothers in formal health systems in low-resource settings was found to be important for overcoming workload through task sharing. They possess the ability to undertake various tasks within facilities and communities aimed at assisting HIV-positive women and their families. EMs frequently serve as auxiliary staff for services facing staffing shortages. Their responsibilities may include administering routine HIV tests typically conducted by healthcare workers (HCWs), offering pre-and post-HIV test counselling, delivering psychosocial support and guidance on treatment adherence, conducting home visits to assess client well-being, sending reminders for clinic appointments, and tracking patients who have been lost to follow up from PMTCT services.

**Reviewers**: have a problem with reference 122 which describes PMTCT in a specific context of a key population, here sex workers.... which are quite different in profile than general population of pregnant women ....

**Response**: *The point is appreciated, and the* reference has been removed.

We appreciate the editor and reviewers for the comments and time taken to read our manuscript. And hope that we have addressed the reviewers’ comments and associated changes in the manuscript to your satisfaction.

Thank you.

Feleke Hailemichael
